# Effect of hypoalbuminemia on drug pharmacokinetics

**DOI:** 10.3389/fphar.2025.1546465

**Published:** 2025-02-20

**Authors:** Ilona Idasiak-Piechocka, Dominik Lewandowski, Wojciech Świgut, Jan Kalinowski, Klaudia Mikosza, Piotr Suchowiejski, Edyta Szałek, Agnieszka Karbownik, Miłosz Miedziaszczyk

**Affiliations:** ^1^ Department of General and Transplant Surgery, Poznan University of Medical Sciences, Poznan, Poland; ^2^ The Student Scientific Society of Poznan University of Medical Sciences, Poznan, Poland; ^3^ Department of Clinical Pharmacy and Biopharmacy, Poznan University of Medical Sciences, Poznan, Poland

**Keywords:** hypoalbuminemia, drug pharmacokinetics, nephrotic syndrome, protein binding, distribution

## Abstract

Hypoalbuminemia, defined as serum albumin levels below 35 g/L, is common in patients with conditions such as nephrotic syndrome, cirrhosis, or sepsis. This review examines the impact of hypoalbuminemia on the pharmacokinetics of selected drugs—such as antibiotics, immunosuppressants, antifungals, or anticonvulsants—emphasizing its role in drug efficacy and safety. Albumin is the main drug transporter and key binding protein, which influences the free drug concentration and drug activity. The review includes all studies available in the scientific literature found in the PubMed, Scopus, and Cochrane databases. The paper emphasizes the importance of therapeutic drug monitoring (TDM) in patients with hypoalbuminemia to avoid subtherapeutic or toxic drug levels. Many drugs need for dose adjustments to achieve therapeutic levels, especially in critically ill patients. The results of studies emphasize the need for individualized dosing regimens based on TDM to optimize drug therapy in patients with hypoalbuminemia. Our review is the first article to summarize the influence of hypoalbuminemia on the pharmacokinetic parameters of drugs and may be a useful tool for clinicians in their daily work.

## Highlights

• Plasma concentration - amount of a drug present in the plasma, the liquid component of blood, at a given time.
• Serum concentration - concentration of a drug in the serum, the fluid portion of blood after clotting factors have been removed.
• Free (drug name) concentration - concentration of the drug in the plasma or serum that is not bound to proteins and is pharmacologically active.
• Total (drug name) - combined amount of both bound and unbound drug in the plasma or serum.
• Total (drug name) concentration - total amount of the drug, including both its protein-bound and unbound forms, in the plasma or serum.
• Unbound (drug name) - portion of the drug that is not attached to plasma proteins and is available to exert a therapeutic effect.
• Unbound (drug name) concentration - concentration of the drug in the plasma or serum that is free from protein binding, reflecting its active form
• Bound (drug name) - fraction of the drug that is attached to plasma proteins, which typically cannot exert an immediate pharmacological effect.
• Bound (drug name) concentration - concentration of the drug in the plasma or serum that is bound to plasma proteins and cannot exert an immediate pharmacological effect.

## 1 Introduction

Hypoalbuminemia is a frequent medical problem that can be caused either by increased protein loss, in such conditions as nephrotic syndrome and protein-losing enteropathy, or reduced protein synthesis in patients suffering from malnutrition and cirrhosis (Ballmer, 2001) It is defined by serum albumin less than 35 g/L. Hypoalbuminemia was found to be one of the indicators of ongoing sepsis and is associated with increased mortality and longer hospital stays regardless of the primary disease (Franch-Arcas, 2001). Although serum albumin level is a nonspecific marker it is a strong predictor of sooner and more frequent readmissions in acute illnesses (Herrmann et al., 1992). In the field of oncology albumin level correlates with negative prognosis and worsened quality of life (McSorley et al., 2017). Hypoalbuminemic state is also present in certain physiological conditions–as pregnancy–associated with increasing permeability or in childhood (Loh and Metz, 2015; Challis et al., 2009).

**FIGURE 1:**
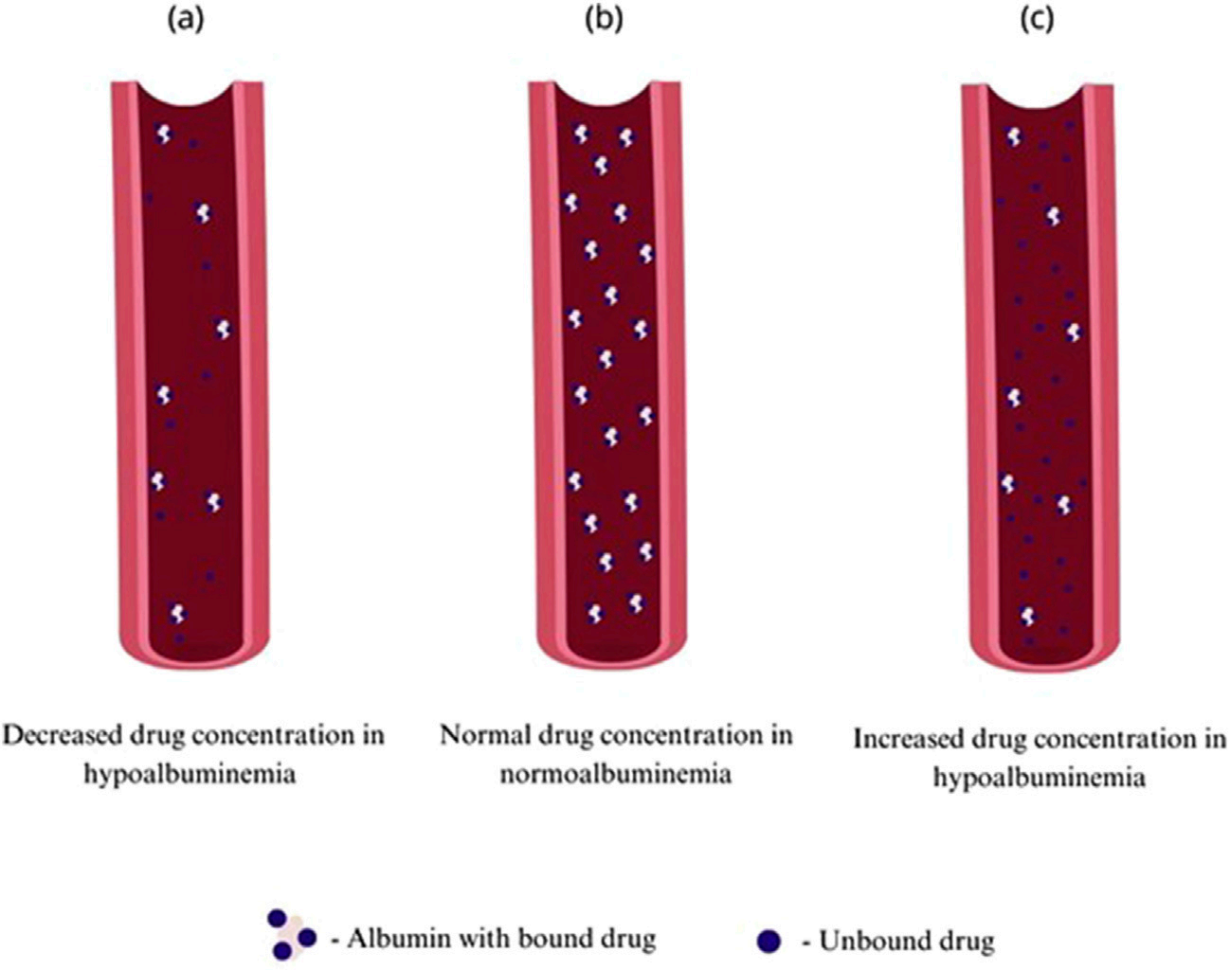
Comparison of unbound drug concentration in patients with normoalbuminemia and hypoalbuminemia conditions: **(A)** decreased concentration of unbound drug in hypoalbuminemia, **(B)** normal concentration of unbound drug in normoalbuminemia, **(C)** increased concentration of unbound drug in hypoalbuminemia.

Human serum albumin is widely known as a main factor maintaining serum oncotic pressure and proper fluid distribution in compartments of an organism, additionally it is involved in maintaining acid–base balance in the plasma. It is also the major carrier for fatty acids, hormones such as thyroxin, cortisol or testosterone and for acidic and neutral drugs working as a delivery system (Fanali et al., 2012). Albumin is synthesized in liver, firstly as a pre-proalbumin converted to proalbumin and then, by Golgi apparatus to its final form–albumin. Its synthesis can be inhibited by inflammatory agents as interleukins or tumor necrosis factor. In physiological conditions kidneys and gastrointestinal tract can cause loss of up to 10% of albumin (Levitt and Levitt, 2016; Cabrerizo et al., 2015). Failure of treatment in such patients can be connected with failure in achieving correct drug concentrations due to insufficient protein binding and drug distribution (Roberts et al., 2013). The French Society of Pharmacology and Therapeutics and the French Society of Anesthesia and Intensive Care Medicine recommends incorporating therapeutic drug monitoring (TDM) such as antibiotics to avoid subtherapeutic drug concentrations due to too low dose values in critically ill patients–very often presenting hypoalbuminemia (Guilhaumou et al., 2019). Changes in protein binding are only relevant for highly-protein bound drugs what is defined as 90%–99.9% protein binding. Another factor influencing pharmacokinetics is non-linear pattern of protein-binding which makes time-concentration profile less predictable (Wright et al., 1996; Deitchman et al., 2018). The aim of TDM is also to avoid toxic drug concentrations that increase the risk of side effects, however only drugs having high extraction ratios (ER) are considered to be susceptible to alterations of plasma protein binding (Smith et al., 2012). Figure 1 presents possible alterations in drugs’ pharmacokinetics caused by hypoalbuminemia, showing that fewer albumin molecules in plasma can lead to higher proportion of the unbound drug, but it is also necessary to take under consideration possibly increased drug metabolism and elimination leading to decreased drug concentration. Albumin can be used as a drug itself in conditions such as hemorrhagic shock, severe burn, cirrhosis with refractory ascites or nephrotic syndrome, however hypoalbuminemia is rather a syndrome than primary process of the disease so it can be used only as symptomatic treatment (Liumbruno et al., 2009). The aim of this review is to assess the effect of hypoalbuminemia on the pharmacokinetics of selected drug classes in patients with low serum albumin concentration.

## 2 Materials and methods

**FIGURE 2:**
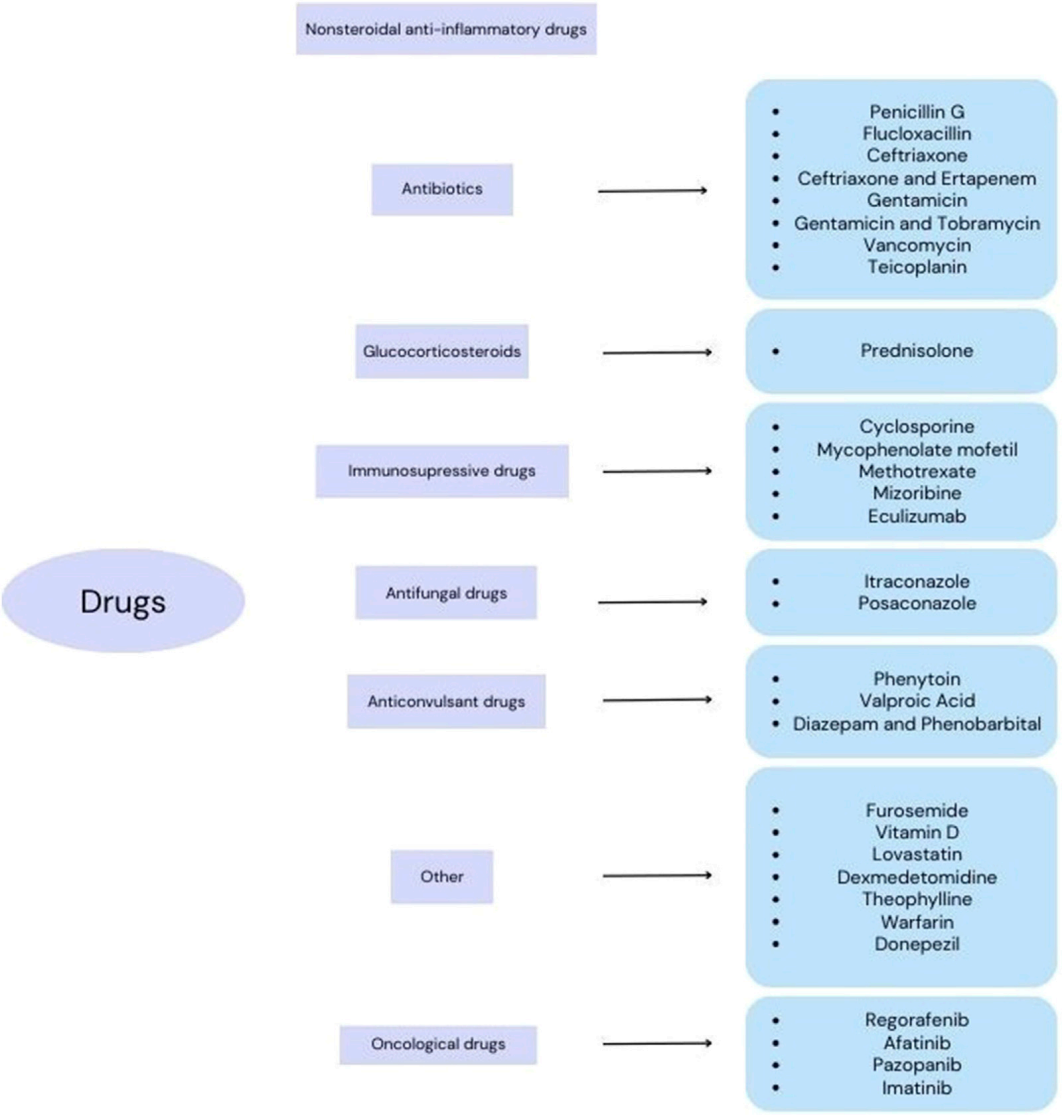
Division of drugs according to the research results available in the scientific literature.

The search strategy included controlled vocabulary and keywords. Four authors independently searched the PubMed, Scopus and Cochrane databases. The main search was done by combining “hypoalbuminemia”, “nephrotic syndrome”, “cirrhosis”, “sepsis”, “epilepsy”, “rheumatoid arthritis”, “infection”, “hyperlipidemia”, “asthma”, “Alzheimer”s disease”, “cancer”and “albumin” with related terms such as “protein binding”, “distribution”, “drug concentration” and “drug monitoring”. The inclusion criterion was the data included in the studies related to the selected groups of drugs. All original studies available in the scientific literature were included in the review. Because of the small number of studies in recent years and difficulties in obtaining essential data the time criterion was not applied. Figure 2 shows the division of drugs according to the results of all studies available in the scientific literature.

## 3 Results

### 3.1 Antibiotics

#### 3.1.1 Time-dependent killing antibiotics (T > MIC)

Antibiotics with time-dependent killing, which means that their efficacy depends on the duration of the concentration above T > MIC (minimal inhibitory concentration), include β-lactams (Szałek et al., 2009).

##### 3.1.1.1 Penicillin G

Penicillin G is a narrow-spectrum antibiotic, used mainly to treat infections caused by Gram-positive bacteria. Due to its low absorption from the gastrointestinal tract (15%–30%), this antibiotic is used intravenously or intramuscularly. It binds to serum proteins in about 45%–68% (Heffernan et al., 2022; Bukkems et al., 2018). Öbrink-Hansen et al. conducted a study on 46 patients undergoing treatment for infective endocarditis (IE), the purpose of which was to determine whether the applied dose of 3 g every 6 h is sufficient to achieve therapeutic levels of penicillin G in patients with, IE and to assess the likely variables that affect inter- and intra-patient variability (Öbrink-Hansen et al., 2017). The mean value of albumin in patients was 26 (Curran et al., 2003; Butterfield et al., 2011) g/dL. 71% of patients achieved 100% fT > MIC, a significant proportion in whom this value was not achieved had additional enterococcal infection. Two samples were collected: after 3 h (sample A, where the assessment goal was 50% fT > MIC) and after 6 h (sample B, where the assessment goal was 100% fT > MIC). Multivariate regression analysis showed that the concentration of penicillin G in sample A at the beginning of the study was significantly correlated with eCLcr (P = <0.0001) and p-albumin (P = 0.02). In subsequent studies, concentrations were determined from 2 to 5 weeks of treatment, and high penicillin levels were significantly correlated with low albumin levels (P = 0.01), as well as with other factors: age, body weight and eCLcr, resulting in higher penicillin levels halfway between doses. No adverse effects have been reported in patients during ongoing treatment hypoalbuminemia is associated with increased levels of unbound drug. Nevertheless, the study showed that in this case, the doses used provide therapeutic concentrations of penicillin G in patients with, IE caused by streptococci and staphylococci, only infection caused by enterococci may require monitoring to determine the treatment regimen.

##### 3.1.1.2 Flucloxacillin

Flucloxacillin is a penicillin antibiotic that exhibits activity against Gram-positive bacteria and is most commonly used in skin and soft tissue infections excluding MRSA. The drug binds to albumin at approximately 93% (Meenks et al., 2023). Yoshiro Hayashi et al. conducted a study in which they aimed to test the utility of monitoring a β-lactam drug in critically ill patients. They analyzed the cases of three patients, where two of them were taking flucloxacillin (Hayashi et al., 2013). The first patient was diagnosed with staphylococcal ventriculitis complicating mastoiditis. The patient was diagnosed with mild renal dysfunction (plasma creatinine stat 134 μmol/L, urea stat 13.2 mmol/L on admission) and hypoalbuminemia (18 g/L). The antibiotic was administered at a dose of 2 g intravenously every 4 h, the regimen used resulted in a drug concentration of 24 mg/L on day 4 (the minimum concentration should be 40–50 mg/L), and the concentration in the CSF was indeterminate (<5 mg/L). The dose was increased to 12 g/day, watcher a concentration of 39 mg/L was achieved, and then increased to 16 g/day, which resulted in a concentration of 51 mg/L on day 9. The second patient was diagnosed with staphylococcal endocarditis complicated by cerebral congestion. The treatment regimen consisted of flucloxacillin i.v. 2 g every 4 h, rifampicin i.v. 600 mg/d and sodium fusidate 500 mg every 8 h via nasogastric tube. The patient was diagnosed with hypoalbuminemia (15 g/L), and it was considered that the distribution volume had doubled therefore a dose of 2 g every 4 h was administrated. Only when renal function deteriorated (secondary drug 147 mg/L) was the regimen changed to 1 g every 4 h. In the following days, RRT had to be included because the patient’s condition was deteriorating, this procedure and the lowering of the dose allowed to reduce the level of flucloxacillin. The two cases presented show that the use of TDM in the treatment of infections in areas with poor penetration (CNS infections) is useful to achieve the intended concentrations without the risk of side effects due to drug toxicity, and prevents suboptimal concentrations between doses (Hayashi et al., 2013).

##### 3.1.1.3 Ceftriaxone

Ceftriaxone is a broad-spectrum antibiotic that belongs to the third-generation cephalosporin group. This antibiotic penetrates tissues well and is therefore often used to treat respiratory, urinary tract, skin and soft tissue infections. It is administered intravenously or intramuscularly, as its bioavailability is less than 1% after oral administration. Ceftriaxone is an antibiotic that binds strongly to proteins 95% and has a long half-life, which translates into a convenient dosing schedule for the drug (Richards et al., 1984; Hartman et al., 2021). Heffernan et al. conducted a study aimed at describing the population pharmacokinetics of total and unbound ceftriaxone in adult critical care patients and optimizing the dosing regimen (Heffernan et al., 2022). They obtained 474 samples from 36 patients (total ceftriaxone = 267, unbound 207). Patients had hypoalbuminemia (mean 23.9 g/L). Based on simulated drug dosing, it was shown that depending on the dosing regimen and renal function, reduced albumin levels were associated with a lower probability of achieving the target (PTA). A 10 g/L reduction in albumin at a given renal function reduces PTA by 20%. It follows that in critically ill patients with hypoalbuminemia and augmented creatinine clearance (ARC), there is a need to increase the dose of ceftriaxone to achieve a Cmin/MIC of >1. The recommended dose for patients with ARC or patients with an isolate infection with a higher MIC (>0.25 mg/L) is 3–4 g per day administered by infusion or in divided doses to achieve a Cmin/MIC of >1. The study’s authors also note caution when administering doses in excess of 2 g per day in patients with renal impairment, as the risk of side effects such as seizures is higher than in patients with normal renal function.

##### 3.1.1.4 Ceftriaxone and ertapenem

Ertapenem is a beta-lactam antibiotic belonging to the carbapenem group. It is used in the treatment of moderate to severe abdominal infections, extramedullary pneumonia, complicated skin infections and acute pelvic infections. This antibiotic is administered intravenously or intramuscularly (after intramuscular administration, its bioavailability is about 90%). Its ability to bind to proteins, mainly albumin, de-creases with increasing plasma concentration of the drug (85%–95% binding to proteins) (Curran et al., 2003). In their study, Dhanani et al. compared the effects of different albumin concentrations on total and unbound ceftriaxone and ertapenem using a sheep model. Eight Sheep were given ceftriaxone at a dose of 40 mg/kg b.w. and ertapenem at a dose of 15 mg/kg b.w. in states of normoalbuminemia, plasmapheresis-induced hypoalbuminemia (26.1 g/L) and hyperalbuminemia induced by administration of 25% albumin solution (35.8 g/L). The study proved that the hypoalbuminemic state was associated with lower AUC 0-24 (in normoalbuminemia 83.6 mg - h/L, in hypoalbuminemia 65 mg - h/L) and higher CL (in normoalbuminemia 21.9 L/h, in hypoalbuminemia 28.5 L/h) of total ceftriaxone concentration than in the other phases and the volume of distribution was higher in hypoalbuminemia 26.8 L compared to 19.9 L in normoalbuminemia. Accordingly, for the concentration of total ertapenem, the hypoalbuminemic state was associated with a lower AUC 0-24 (in normoalbuminemia 26.9 mg - h/l, in hypoalbuminemia 24.8 mg - h/l) and a higher CL (in normoalbuminemia 25 L/h, in hypoalbuminemia 28.8 L/h). In hypoalbuminemia for unbound ceftriaxone concentration, AUC 0-24 was significantly reduced (normoalbuminemia 68 mg - h/l, hypoalbuminemia 51 mg - h/l), CL was significantly increased in hypoalbuminemia (normoalbuminemia 27 L/h and 36.3 L/h) and volume of distribution was higher in hypoalbuminemia 36.6 L compared to 23.5 L in normoalbuminemia. Correspondingly, for unbound ertapenem concentration, the values were (normoalbuminemia 16 mg - h/l, hypoalbuminemia 14.5 mg - h/l) and CL was increased in hypoalbuminemia (normoalbuminemia 42.3 L/h and 49.9 L/h), volume of distribution was 38.4 L in hypoalbuminemia compared to 42.1 L in normoalbuminemia. The study found no significant difference between ceftriaxone and ertapenem binding to proteins in normo and hypoalbuminemia. A limitation of the study was that the difference in albumin concentration was too small when comparing normo and hypoalbuminemia. Unbound drug concentrations are often a surrogate for determining tissue drug concentrations; in the study, unbound ceftriaxone concentrations were significantly lower in the hypoalbuminemic phase (Dhanani et al., 2020). For ertapenem, although the effects were similar, they did not achieve statistical significance which may have been due to a limitation of the study (possibly too small a difference in albumin concentration or too small a test sample size. Hypoalbuminemia is common in critically ill patients, the above study shows that it is correct to use higher doses in a group of patients who have hypoalbuminemia. However, the limitation is the non-linearity of protein binding does not allow predicting the exact concentration of the drug, so it is recommended to monitor the drug by direct measurement of drug concentrations.

#### 3.1.2 Concentration-dependent killing antibiotics (Cmax/MIC)

Aminoglycosides are a group of antibiotics whose activity of action depends on the amount of dose, so they show the greatest effectiveness when administered at a high dose. The Cmax/MIC for aminoglycosides should be above 8. Drugs in these groups show a post-antibiotic effect (PAE), if high enough doses are administered these drugs are still effective even though the tissue and plasma concentrations are below the MIC between doses, due to the occurrence of PAE it is possible to have a longer interval between doses without exposing the drug to lack of effect (Szałek et al., 2009).

##### 3.1.2.1 Gentamicin

Gentamicin is an antibiotic used to treat mainly infections caused by aerobic bacteria. It is used parenterally because absorption from the intestinal lumen is low after oral administration. The antibiotic binds to proteins at about 15% (Bailey and Briggs, 2004). Bukkems et al. conducted a study aimed at evaluating the predictive efficacy of a pharmacokinetics model in critically ill patients in intensive care units (ICUs) (Roberts et al., 2013). Patient results were obtained from two institutions collected from AMC (Academic Medical Center, Amsterdam) included 192 samples from 66 patients (albumin concentration 25 (Öbrink-Hansen et al., 2017; Meenks et al., 2023; Hayashi et al., 2013; Richards et al., 1984; Hartman et al., 2021; Curran et al., 2003; Dhanani et al., 2020; Bailey and Briggs, 2004; Etzel et al., 1992; Liu et al., 2021; Butterfield et al., 2011; He et al., 2016; Bailey et al., 1991) g/L), a collection from CHU Nîmes (Centre Hospitalier Universitaire de Nîmes) included 230 samples from 50 patients (albumin concentration 28.7 (25.1–32.8) g/L). Patients received gentamicin at a dose of 5 mg/kg for suspected septicemia and 3 mg/kg for endocarditis. Based on data from the AMC, which obtained more data on albumin levels in patients, a negative correlation was shown between gentamicin distribution volume and serum albumin levels. Albumin levels below 20 g/L are associated with lower and subtherapeutic Cmax. Due to the paucity of concentration measurements with CHU, the relationship was not confirmed. Although the study showed that the presented pharmacokinetic model cannot be applied to other CHUs, it provided important information on how to exercise caution when dosing aminoglycosides in patients with hypoalbuminemia. The authors of the study suggest taking into account albumin levels in gentamicin dosing and testing Cmax at the beginning of treatment in patients with albumin levels below 20 g/L or using a dose above 6 mg/kg with in patients with albumin levels below 20 g/L in the absence of TDM (Bukkems et al., 2018).

##### 3.1.2.2 Gentamicin and tobramycin

Tobramycin is an antibiotic belonging to the aminoglycoside group, which is mainly used to treat infections of the lower respiratory tract, urinary tract, skin, eye, bone and infections in patients with coexisting cystic fibrosis. The drug can be administered intravenously, by inhalation or as eye drops; when administered intravenously, tobramycin is poorly absorbed. The drug binds to proteins at about 30% (Bailey and Briggs, 2004).Etzel et al. conducted a study to evaluate the pharmacokinetics of gentamicin and tobramycin in patients without pleural effusion (n = 1,049), (mean albumin concentration in this group was 2.8 ± 0.7 g/dL) and with pleural effusion and concomitant hypoalbuminemia (n = 260) (mean albumin concentration 3.2 ± 0.7 g/dL). Patients received aminoglycoside doses such that the 1-h peak concentration was 5–10 mg/dL and the minimum con-centration was more than 2 mg/dL, regardless of the dosing interval (Etzel et al., 1992). Multiple linear regression analysis showed significant effects on the volume of distribution of aminoglycosides and the associated half-life in the elimination phase of the drug: higher age (P = 0.04), low serum albumin concentration (P = 0.0001), total patient weight (P = 0.0001) and the presence of pleural effusion on X-ray (P = 0.008), while total amino-glycoside clearance did not differ significantly between patients (P > 0.4). The analysis results presented here indicate that patients with pleural effusion, which is associated with hypoalbuminemia, may require higher doses of aminoglycosides such as gentamicin and tobramycin to achieve therapeutic drug concentrations. The study authors recommend monitoring aminoglycoside levels in patients with pleural effusion to pre-vent subtherapeutic drug concentrations (Etzel et al., 1992).

#### 3.1.3 Concentration-dependent antibiotics with a time-dependent component (AUC24/MIC)

Glycopeptides are a group of antibiotics that exhibit efficacy from the drug con-centration and the time at which the drug concentration is above the MIC. The high efficacy of these drugs is associated with the administration of high doses with a long half-life, with an increase in the frequency of doses if the substance used has a short half-life, or with administration by prolonged infusion (Liu et al., 2021).

##### 3.1.3.1 Vancomycin

Vancomycin belongs to the group of glycopeptide antibiotics. It is used to treat sepsis, infective endocarditis, skin and bone infections (intravenous administration) and to treat pseudomembranous enteritis caused by *Clostridium difficile* bacteria (oral administration). The drug binds to serum proteins at about 50% (Butterfield et al., 2011). He et al. conducted a study to evaluate pharmacokinetics in patients with severe acute pancreatitis (SAP) (n = 67) (He et al., 2016). The mean dose received by patients was 15.0 ± 3.7 mg/kg. Patients with SAP showed significantly lower vancomycin levels (6.1 ± 3.0 mg/L; P < 0.0001) with a volume of distribution of 62.9 ± 16.5 L and a CL of vancomycin of 9.9 ± 3.5 L/h. The study authors observed a strong correlation between minimum vancomycin concentration and age (P = 0.004), albumin (P = 0.02) and treatment duration (P < 0.0001). A stepwise regression analysis was also performed on the basis of which information was obtained that duration was the most important variable (R2 = 0.456), then patients were divided into an early group (treatment duration <4 weeks) and a late group (treatment duration >4 weeks).Vancomycin clearance in the early group was significantly higher than in the late group (10.8 ± 3.3 vs 6.3 ± 1.5 L/h). Based on the study, it was shown that standard doses of vancomycin may not be sufficient for patients with SAP accompanied by hypoalbuminemia to achieve the recommended 24 h AUC/MIC value (for *S. aureus* = 400) and higher doses should be used. Although the statistical significance of lower albumin levels has been demonstrated, the study authors suggest further investigation on this issue (He et al., 2016).

##### 3.1.3.2 Teicoplanin

Teicoplanin, like vancomycin, belongs to the glycopeptide antibiotics, similar to vancomycin it also has a similar mechanism and spectrum of action. Parenteral it is used in the treatment of skin, bone, soft tissue infections, pneumonia, infective endocarditis and orally it can be used as an alternative to vancomycin in the treatment of colitis caused by *C. difficile* infection. Teicoplanin binds strongly to proteins about 90% (Bailey et al., 1991). Roberts et al. studied the variability of teicoplanin’s binding to proteins and the effectiveness of the therapy in monitoring the drug in critically ill patients (n = 13). Patients received daily doses ranging from 2.7 to 20 mg/kg. The range of albumin levels in the patients studied was 17.5–34 g/L. The lower therapeutic range was 10–20 mg/L, which 42% of patients achieved, and the higher range was 10–30 mg/L, which 58% of patients achieved, and the dose used correlated with total teicoplanin concentration (R2 = 0.5996). Among the patients studied, free teicoplanin plasma concentrations ranged from <0.1 mg/L to 10 mg/L (average dose) and 0.1 mg/L to 4.5 mg/L (minimum). The study showed significantly higher unbound fractions in patients with lower albumin levels (P = 0.0078). The authors suggest that for this group of patients, the required minimum daily dose should be 6 mg/kg to achieve a minimum concentration (≥10 mg/dL) and recommend considering a saturating dose (12 h for the first three doses) to avoid subtherapeutic concentrations of the drug. Roberts et al. infer from their results that hypoalbuminemia in critically ill patients is associated with a very high variability in the binding of teicoplanin to proteins, which supports the use of monitoring free teicoplanin concentrations instead of total concentrations in TDM (Roberts et al., 2014). At the same time, they note the need to confirm this in a prospective study.

### 3.2 Glucocorticosteroids

Glucocorticosteroids are some of the most commonly used anti-inflammatory drugs today, they are derivatives of steroid hormones produced in the striatal layer of the adrenal cortex.

#### 3.2.1 Prednisolone

Prednisolone, which belongs to the group of glucocorticosteroids, has a wide range of action, and is used in the treatment of adrenal insufficiency, inflammation, hematopoietic conditions and cancer. This drug can be used intravenously, orally and rectally. The bioavailability of prednisolone after oral administration reaches about 70%. Binding to proteins is variable, ranging from 65% to 91% in healthy individuals (Pickup, 1979; Ferry et al., 1988). Bergrem et al. compared three groups: nephrotic syndrome patients with normal serum creatinine levels (n = 7), patients on dialysis (n = 7) and a control group (n = 12) in terms of pharmacokinetics and prednisolone binding to proteins. All received 20 mg of prednisolone sodium succinate. Patients with nephrotic syndrome had normal serum creatinine levels and normal liver function tests. The authors of the study showed that patients with nephrotic syndrome relative to the control group had significantly lower peak serum prednisolone concentrations by 24% (P < 0.05), (in patients with nephrotic syndrome Cmax = 366 ng/mL, and in patients with normoalbuminemia Cmax = 481 ng/mL), significantly higher volume of distribution at steady state by 39% (P < 0, 05), (in nephrotic syndrome patients VD = 43.5 L, and in normoalbuminemic patients 26.7 L), significantly smaller area under the curve of total prednisolone concentration over time by 31% (P < 0.05), (in nephrotic syndrome patients AUC = 1787 ng*h/mL, and in normoalbuminemic patients AUC = 2,578 ng*h/mL), a significantly higher total prednisolone clearance by 32% (P < 0.05), a significantly higher free prednisolone fraction by 14%, and a smaller area by 13% under the curve for free prednisolone concentration. Bergrem et al. showed, based on their study and the in-formation cited above, that a reduction in prednisolone dose may not be necessary if renal and hepatic function is normal, as dis-orders affecting these organs can affect clearance and binding to proteins, confounding the effect of hypoalbuminemia alone in nephrotic syndrome (Bergrem, 1983).

### 3.3 Immunosupressive drugs

Immunosuppressive drugs are medications that modulate or suppress the immune response, often to prevent organ rejection in transplant patients or to manage autoimmune diseases. The dosing and efficacy of immunosuppressants depend on achieving plasma concentrations within the therapeutic window to prevent toxicity or graft rejection. Many of these drugs require close monitoring due to their narrow therapeutic index and potential for adverse effects such as nephrotoxicity, hypertension, or increased risk of infections.

#### 3.3.1 Cyclosporine

Cyclosporine is an immunosuppressive drug used in various inflammatory conditions such as rheumatoid arthritis, ulcerative colitis, membranous glomerulonephritis and to prevent transplant rejection. It helps reduce the doses of steroids and their adverse reactions. Cyclosporine is also used in steroid-resistant nephrotic syndrome. On the other hand, it can cause a variety of side effects by itself, even within its therapeutic range levels. Cyclosporine is lipophilic (Barbarino et al., 2013) Around 50% of the administered dose is found in erythrocytes’ cytoplasm (Freeman, 1991; Reinhart WH, 1993). 90% of plasma concentration is bound to proteins, mainly lipoproteins. In healthy individuals 50%–60% of the plasma CsA is bound to HDL, 20%–30% to LDL, 10%–25% to VLDL and 10%–15% to non-lipoprotein proteins. Hyperlipidemia increases a fraction bound to LDL and VLDL without altering the amount bound to non-lipoprotein proteins (Abdel-Mottaleb et al., 2023). A study conducted by Jacqz-Aigrain et al. showed no correlation between serum albumin and cyclosporine clearance. However, a relation between serum albumin and cyclosporine free fraction was not determined in the study. Other factors that may coexist with hypoalbuminemia should be taken into account. Hematocrit was found to affect cyclosporine clearance, cholesterol level was interestingly not. A significant correlation between creatinine clearance and cyclosporine clearance in the study is worth noticing, however probably coincidental (Jacqz-Aigrain et al., 1994).

A number of studies (Jacqz-Aigrain et al., 1994; Takeda et al., 2007; Lindholm et al., 1990) indicate the changes in absorption may be the cause of significant differences between achieved drug levels after oral intake. The absorption of cyclosporine occurs mainly in the intestine (Barbarino et al., 2013; Forsythe and Paterson, 2014) and is largely affected by bile acid (Zhao et al., 2010), possibly due to enterohepatic recirculation (Ferry et al., 1988). Thus therapeutic drug monitoring seems to be mandatory (Naito et al., 2008; Faulds et al., 1993; Saito et al., 2014) for all the patients treated with cyclosporine, not only those with hypoalbuminemia. It is also worth noticing that high dose CsA can inhibit hepatic albumin synthesis, leading to hypoalbuminemia (Jeon and Kim, 2011).

Although cyclosporine may be the cause of a variety of side effects, their use may actually decrease pharmacotherapy’s adverse reactions frequency by reducing the use of steroids, as in the study conducted by Shirai et al. (Shirai et al., 2018). The risk to benefit ratio and adequate dosage should always be individually evaluated.

#### 3.3.2 Mycophenolate mofetil

Mycophenolate mofetil (MMF) is an immunosuppressive drug, usually combined with steroid and/or cyclosporine to prevent organ rejection and as a treatment of various diseases with nephrotic syndrome. It is a prodrug of mycophenolate acid (MPA), a reversible inhibitor of inosine monophosphate dehydrogenase (IMPDH) (Allison, 2005). Absorption in the small intestine after oral intake and metabolism is rapid and not affected by food, reaching maximum plasma concentration of MPA within 60–90 min 97% of plasma MPA is bound to protein, mainly albumin (Park, 2011).

A number of studies (Naito et al., 2008; Nishimura et al., 2022; Reine et al., 2013) confirm a significant negative correlation between albumin and MPA free fraction, but a positive correlation between albumin and MPA AUC 0-12 h (Nishimura et al., 2022). Thus MPA free fraction seems to be more suitable for therapeutic drug monitoring than total MPA concentration, but as demonstrated in the research conducted by Reine et al. (Reine et al., 2013), both total MPA and free MPA may be equal predictors of the immunosuppressive response with albumin level within 25–35 g/L range examined in the study. In cases of severe hypoalbuminemia however, total MPA concentrations may stay low and not increase (Nishimura et al., 2022).

Other factors influencing MMF and MPA pharmacokinetics include body weight (Naito et al., 2008), kidney function (Reine et al., 2013), liver metabolism, genetically determined IMPDH enzyme activity, enterohepatic recirculation (which may be inhibited by Cyclosporine A coadministration) and possibly also enzyme induction as a result of MMF administration (Sanquer et al., 1999; Wang et al., 2007). Hematological abnormalities, gastrointestinal symptoms are possible side effects and signs of overdose (Park, 2011; Alex et al., 2014; Parfitt et al., 2008). Narrow therapeutic index (Reine et al., 2013), high variability in pharmacokinetics between patients and within subjects over time support the idea of implementing TDM when MMF is administered (Reine et al., 2013; Bullingham et al., 1998; Pisupati et al., 2005). TDM based on MPA AUC 0–12 h is the most accurate way to monitor and adjust dosage, however in patients with nephrotic syndrome TDM based on MPA C through appears to be accurate enough and efficient, as it does not require multiple blood samples within 12 h (Resztak et al., 2021).

#### 3.3.3 Methotrexate

Methotrexate (MTX) is an immunosuppressive and antineoplastic agent that acts as antimetabolite, blocking dihydrofolate reductase and cell division. The drug has a narrow therapeutic index, being nephrotoxic, myelotoxic, hepatotoxic and causing gastrointestinal symptoms. Only around 50% of methotrexate is bound to plasma proteins. Volume of distribution is 1L/kg (Inoue and Yuasa, 2014). However, as shown in a study by Reiss et al. (2016), hypoalbuminemia is altering MTX pharmacokinetics as there is a significant negative correlation between albumin level and MTX clearance time, as patients with albumin level below 3.4 g/dL had median time to MTX clearance of 72 h compared to 96 h of non-hypoalbuminemic patients. The reason for this may be a decrease in oncotic pressure, leading to third spacing by shifting fluid into interstitial spaces. This would result in MTX being accumulated in the third space and slowly released over time. The relation between albumin levels and methotrexate free fraction was not determined.

Reiss et al. (2016) showed few differences in toxicity between patients with hypo- and normoalbuminemia, but the sample size included only 32 patients with hypoalbuminemia and other medications were administered, which may have affected the results. Barakat et al. (2022) and Khera et al. (2022) showed statistically significant positive correlation between hypoalbuminemia and MTX toxicity in children with acute lymphoblastic leukemia. Hence therapeutic drug monitoring and leucovorin or folinic acid administration (Ackland and Schilsky, 1987; Treon and Chabner, 1996) seem useful.

### 3.4 Nonsteroidal anti-inflammatory drugs (NSAIDs)

NSAIDs are a widely used class of drugs that reduce inflammation, pain, and fever. Their primary mechanism involves inhibiting cyclooxygenase (COX) enzymes, which are critical for the synthesis of prostaglandins involved in inflammation and pain signaling. Long-term or high-dose use of NSAIDs is associated with adverse effects such as gastrointestinal ulceration, renal impairment, and increased cardiovascular risk. To optimize their therapeutic effect while minimizing risks, they are often prescribed at the lowest effective dose for the shortest possible duration. They are all highly protein bound, with fraction bound to plasma albumin exceeding 95%. This causes important medical implications, as they can displace other drugs from albumin binding sites, increasing toxicity (Weinblatt, 1989). Hypoalbuminemia may also increase the NSAIDs’ free fraction, resulting in greater efficacy. As shown in several studies (Van den Ouweland et al., 1986; Van den Ouweland et al., 1988), patients with rheumatoid arthritis, whose albumin levels fluctuate greatly depending on a disease activity, present variable free fraction of naproxen. Treated with 500 mg of twice daily, during active disease peak unbound drug concentration (Cmax) was 0.73 mg/L and AUC0-12 h of unbound drug was 2.94 mg*h/l, compared to Cmax = 0.32 mg/L and unbound drug AUC0-12 h = 1.94 mg*h/l at the time of improvement. During the relapse, albumin levels drop, naproxen free fraction levels increase, which might be even beneficial. Therapeutic drug monitoring is not recommended, but when taken chronically, periodic examination of the patient in search of side effects is valuable. It is worth noting that NSAIDs used long-term may cause small intestine inflammation as their adverse reaction in adults and children, resulting in protein loss and hypoalbuminemia (Boodhan and Friedman, 2009).

### 3.5 Antifungal drugs

Antifungal drugs are agents used to treat fungal infections, targeting the unique components of fungal cells. The efficacy of these drugs depends on both the drug concentration and the duration of exposure above the minimal inhibitory concentration (MIC). For instance, azoles inhibit ergosterol synthesis. These drugs are often associated with dose-dependent toxicities such as hepatotoxicity requiring careful monitoring during therapy.

#### 3.5.1 Itraconazole

Itraconazole (ITZ) is a highly lipophilic triazole antifungal agent used to treat various fungal infections, such as blastomycosis and onychomycosis. It has a volume of distribution exceeding 700 L in adults and extensively distributes into tissues, with organ concentration reported to be 2-3 times higher than corresponding plasma concentration. ITZ is extensively metabolized in the liver, primarily into hydroxyitraconazole, which has been shown to exude comparable antifungal activity to its’ parent compound (De Beule and Van Gestel, 2001). Mochizuki et al. reported a case of a 75-year-old man suffering from Aspergillus sp. induced hypertrophic pachymeningitis, which was unsuccessfully treated with antifungal agents until 200 mg ITZ daily was issued. Abnormally low levels of serum ITZ were reported on day 1, as indicated by peak plasma concentration Cmax = 93.2 vs. 215.6 ng/mL; mean ITZ area under the curve of the serum level was also decreased AUC0-24 = 999 vs. 4,142 ng h/mL compared to healthy subjects. Hydroxy-itraconazole reached Cmax = 159.4 ng/mL and AUC0-24 = 1,391 ng h/mL. These findings were associated with hypoalbuminemia, which was diagnosed on day 2, as the patient suffered renal insufficiency because of previous amphotericin B treatment. Plasma albumin level was 2.8 g/dL at ITZ initiation and increased to 3.9 g/dL by the end of therapy. Researchers believe this to be the reason for plasma drug concentrations reaching 452 ng/mL at 36th month of treatment as albumin normalized. ITZ binds to protein in 99.8%, with albumin being the main binding agent (99.6% for hydroxy metabolite), which could cause an increase in the unbound form. The resulting decrease in total drug concentration in blood would be the result of blood vessel permeation and high tissue concentration. This explanation remains unconfirmed as neither unbound ITZ serum concentration nor tissue concentrations were measured. The infection did not recur, and no adverse effects were observed which would suggest that administrated dose was enough to exert therapeutic effect, attributing lower than expect ITZ levels to insufficient measuring techniques rather than decreased drug concentration. This remains consistent with previous findings as the researchers deny the patient taking H2 blockers or enzyme inducers that have been shown to decrease serum ITZ concentration. Route of administrations should be also factored in as a possible explanation. ITZ bioavailability when given orally stands at 55%, yet requires acidic gastric environment that, as researchers suggest, could have been increased due to age and low fat meal prior to drug administration (Mochizuki et al., 2000).

#### 3.5.2 Posaconazole

Posaconazole is also a triazole antifungal drug, used to treat invasive *Candida* sp. and Aspergillus sp. infections in immunocompromised patients. It is present in plasma primarily in unmetabolized form, which is highly protein bound (>98%), predominantly to albumin. The metabolism of posaconazole is mediated predominantly through uridine diphosphate-glucuronyltransferase enzyme pathways. Excreted metabolites account for only 17% of administered dose and are eliminated with a mean half-life of 35 h (Li et al., 2010). A recent report in Open Forum Infectious Diseases mentions a link between hypoalbuminemia and the occur-rence of subtherapeutic posaconazole concentrations, with 22.7% of patients failing to achieve the trough concentrations desired for prophylaxis, and 50% failing to achieve concentrations of at least 1.25 μg/mL for therapy. David et al. point to unbound concentration remaining unchanged despite albumin decrease as it is governed by intrinsic clearance and unbound volume of distribution. The unbound drug remains in equilibrium with the bound drug, thus the capacity to bind the drug is reduced proportionally to the reduction in albumin concentration. The more extensive the protein binding, the greater its effect on the pharmacokinetics of the total drug, particularly on total clearance and volume of distribution (Nix et al., 2024) Maleki et al. reported a case of a 57-year-old male suffering from acute myeloid leukemia and pulmonary aspergillosis. Patient exhibited low albumin levels throughout hospitalization consistently averaging 25 g/L. The cause of hypoalbuminemia was not specifically disclosed by authors but could result from aforementioned comorbidities. Treatment with posaconazole was issued using tablets of 300 mg/d for 5 days, with initial steady state posaconazole trough level of 0.84 mg/L (target >0.70 mg/L). After a spike in bilirubin was noted (36 mm/L) total serum posaconazole concentration dropped to subtherapeutic 0.4 mg/L and a dosage increase on day 5 through 12–400 mg/d commenced. Failure to achieve target levels and treatment success eventually led to a replacement of posaconazole with voriconazole, which elicited full recovery. Researchers conclude that failure to achieve target drug levels and therapeutic effect of therapy can be attributed to hypoalbuminemia and hyperbilirubinemia (Maleki et al., 2018). Faster posaconazole elimination can be expected, as low albumin level increases unbound drug fraction available for metabolism and prolonged state of high bilirubin concentration can lead to increased glucuronidation through pathway upregulation. Bilirubin may compete for binding to serum albumin further expanding unbound drug fraction. These changes to drug concentration cannot be confirmed since unbound drug concentration was not measured in this study.

### 3.6 Anticonvulsant drugs

Anticonvulsant drugs are used to prevent or reduce the frequency of seizures in individuals with epilepsy or other seizure disorders. The choice of anticonvulsant depends on the type of seizures, patient factors, and drug pharmacokinetics. These drugs require individualized dosing and titration to achieve therapeutic plasma levels while avoiding adverse effects such as sedation, dizziness, or long-term complications like hepatic dysfunction.

#### 3.6.1 Phenytoin

Phenytoin is an anticonvulsant with a narrow therapeutic range of 10–20 mg/L, which is heavily influenced by comorbidities and albumin levels as it binds to plasma protein in 90%. After intravenous administration half-life of phenytoin ranges from 10 to 15 h, with the majority being excreted as inactive metabolites in bile. Toxicity affects mainly the nervous and cardiovascular system, owing to antiarrhythmic activity. Drug monitoring is recommended as neurotoxicity is dependent on serum concentration, which can result in mild nystagmus, tremors and nausea or at higher levels produce coma and seizures. Tbler et al. described a case of a 25 years old HIV-positive woman with focal segmental glomerulosclerosis and hypoalbuminemia, who experienced severe phenytoin toxicity following hospital admission. She received an initial drug loading phase of 800 mg iv, followed by a regime of 400 mg/d iv phenytoin due to generalized clonic-tonic seizure. The patient exhibited marked lethargy, was unresponsive to deep pain and experienced bilateral clonus and tremors. Laboratory found free phenytoin concentration of 4.9 μg/mL (norm 1–2 μg/mL) with total concentration of 10.9 μg/mL and plasma albumin of 0.2 g/dL. Phenytoin doses were subsequently withheld and after 24 h the patient was alert, with no clinical signs or symptoms of phenytoin toxicity or seizure activity. Researchers remark that AIDS, with associated renal abnormalities or hypoalbuminemia, may increase the risk for elevated free fractions of phenytoin (Tbler et al., 1990).

In another study, phenytoin caused marked clinical toxicity in a 73-year-old man treated for post-herpetic neuralgia. Patient reported ataxia and drowsiness, while receiving a dose of 400 mg daily. Administration route was not specified. Measured serum total phenytoin concentration was Cmax = 58 μmol/L (N.R. 40–80 μmol/L), with serum free phenytoin concentration increased to 10.2 μmol/L (N.R 4–8 μmol/L) and serum albumin of 23 g/L. Low serum albumin was attributed to undernutrition. After lowering dosage to 250 mg/d free phenytoin concentration normalized and symptoms subsided (Fulton et al., 1988).

Seizures are a common symptom of central nervous system disease in patients with HIV infection and many remain on anticonvulsant therapy, as recurrence is high if left untreated. Free phenytoin serum concentration proves valuable in treatment of hypoalbuminemia patients, yet it could be difficult to access and time consuming, which is not efficient for bedside dose titration. Researchers suggest usage of the Sheiner Tozer algorithm for calculating free drug concentration. A study of 23 patients with low plasma albumin measured free PHT fraction to be 1.1 mg/L (±0.5 mg/L); which was comparable to calculated free phenytoin fraction with Sheiner Tozer algorithm (1.2 ± 0.7 mg/L). Authors of the study point out that the formula frequently underestimates, rather than overestimates the measured free PHT in relation to therapeutic ranges, but proves useful when the latter measurement is not feasible accessible (Tobler et al., 2016).

Hypoalbuminemia is also frequent in patients with HIV infection, which may complicate drug concentration readings. Serum phenytoin concentrations were studied in 21 patients with AIDS. Mean blood albumin was low (3.2 ± 0.8 g/dL) and was found to be associated with increased measured free phenytoin concentration. However, free phenytoin concentration was lower than calculated using Sheiner Tozer algorithm, further encouraging free concentration measurements (Burger et al., 1994).

Similarly, Krasowski et al. concluded that measured free phenytoin concentration should be obtained where possible to guide dosing and adjusted concentration can supplement total concentration in patients with low plasma albumin (Krasowski and Penrod, 2012).

#### 3.6.2 Valproic acid

Valproic acid (VPA) is an anticonvulsant mainly used in controlling complex partial and simple or complex absence seizures but has also found usage in treatment of bipolar disorder as well as migraine. It has a half time of 13–19 h and is eliminated through hepatic metabolism in 30%–50% In blood it binds to protein in 80%–95%, primarily to albumin. This affinity remains linear at 40 μg/mL, and changes to nonlinear at higher concentrations. In hypoalbuminemia patients, VPA binding may decrease, in which case a patient can experience toxic effects although the total concentration is within the therapeutic range. Symptoms of overdose include somnolence, heart block, deep coma, and hypernatremia, with fatalities being reported in extreme cases. However, patients have recovered from valproate serum concentrations as high as 2,120 μg/mL. Dautzenberg et al. described a case of 66-year-old women with bipolar disorder, who suffered severe reversible cognitive impairment because of VPA toxicity. She was initially dosed 300 mg/d due to hypomania, with gradual increase over a course of 49 days to maximum recommended dose of 2,500 mg/d as mania developed. This resulted in total valproic acid serum concentration (tVPA) of 30 mg/L (therapeutic: 40–120 mg/L), which prompted use of 4,000 mg/d VPA, as the measured value was believed to be subtherapeutic and mania worsened. Her Montreal Cognitive Assessment score had declined to 15 of 30 from 24 on admission, and Mini-Mental State Examination was 20 of 30 on 109th day of hospitalization. Eventually her cognitive function severely deteriorated with conative function deficits and activities of daily living dependency comparable with Geriatric Deterioration Scale 4. The patient became apathetic, could barely be motivated to eat or drink, and was in need of a wheelchair. These findings were retrospectively found to coincide with significantly raised free serum concentration of VPA reaching 37.8 mg/L (reference range, 4–12 mg/L) at with total fraction of VPA to marked hypoalbuminemia in course of membranous glomerulopathy. There was a time correlation with the tVPA, free concentration and the severity of cognitive impairment, that started at 4,000 mg VPA, and deteriorated as the patient’s albumin dropped from stable 23–26 g/L to 14 g/l. As VPA was withdrawn, she switched to a hyperactive delirium and suffered a seizure, which was successfully treated with 5 mg of olanzapine. Dechallenge of VPA gradually resulted in a continuous revitalization as her cognitive and cognitive functioning recovered to pre-VPA levels (Dautzenberg et al., 2018).

Similar findings were shared in a study of 15 intensive care unit patients with albumin level below 4 g/dL treated with VPA for seizures, myoclonus, bipolar disorder, schizophrenia and refractory agitated behavior. At a daily dose of 3 g, the free fraction of VPA was 48%, with a range of 15%–89%, compared to reference 5%–10%. The researchers note that protein binding within the cohort was highly inconsistent and could not be predicted using tVPA, even when correcting for albumin. Measured tVPA was 52 mg/L (therapeutic: 29–65 mg/L), presenting low or reference range values of 22–92 mg/L, despite high free serum valproate concentrations that were often above the reference range at 20 (therapeutic: 18–30) mg/L (range: 11–37 mg/L). 10 patients (68%) experienced adverse drug events, seven hyperammonaemia, two elevated transaminases and five thrombocytopenia (Riker et al., 2017). Gideal et al. also reported on a patient receiving 4 g of VPA with albumin concentration of 33 g/L, that produced difficulty in walking and involuntary muscle jerks. tVPA concentration was 103 μg/mL and free plasma concentration 26.8 μg/mL. Symptoms resolved once dosage reduction was introduced (Gidal et al., 1993).

#### 3.6.3 Diazepam and phenobarbital

Diazepam is a long-acting benzodiazepine used to treat panic disorders, severe anxiety, alcohol withdrawal, and seizures. When ingested it is absorbed rapidly in more than 90% and reaches peak serum concentration within 1.5 h. Diazepam binds to plasma protein in 98%–99%, mainly to albumin. In the liver it undergoes metabolization to desmethyldiazepam, temazepam and oxazepam, which are all pharmacologically active forms and extend drugs’ activity due to long half-life. Similarly, Phenobarbital is also a long-lasting anticonvulsant used in the treatment of all types of seizures. It has a half-life of 79 h yet binds to protein in only 20%–45%. It is counter advised to combine phenobarbital and diazepam as they both exert inhibitory effects on the central nervous system, which could lead to respiratory depression.

Nonetheless, usage of both diazepam and phenobarbital was reported by Traeger et al. where combination of these drugs induced coma, which occurred in the treatment of alcohol withdrawal seizures. The patient was a 52-year-old female alcoholic suffering from severe liver disease and had albumin of 4 g/dL on admission that dropped to 2.7 while hospitalized. She received 660 mg iv phenobarbital on day 1 and 2, followed by 60 mg every 12 h on day 3 through 12; diazepam was administered on day 2 through 5 in increments 5–10 mg iv for a total dose of 152.5 mg. On day 12 the patient was comatose and orally activated charcoal was administered subsequently. Measured day 2 phenobarbital serum concentration was 19,4 mg/L (N.R 15–40 mg/L) and remained consistent until administration of oral activated charcoal on day 13. Diazepam serum concentration increased throughout admission, peaking at 0.33 mg/L (N.R 0.1–0.8 mg/L), with desmethyldiazepam concentration of 1.1 mg/L (N.R 0.2–0.65). Serum free fraction of both drugs was not measured but is believed to be increased as a result of low serum albumin presented by patients. This remains to be the main contributing factor in observed toxicity as authors suggest (Traeger and Haug, 1986).

### 3.7 Others

#### 3.7.1 Furosemide

Furosemide is a competitive inhibitor of sodium-potassium-chloride cotransporters in the proximal and distal tubules and the thick ascending loop of Henle. It pro-motes diuresis by increasing the excretion of water and electrolytes. It binds primarily to serum albumin. Furosemide is bound in 91%–99% in healthy subjects, the unbound fraction is around 2%–4%. Furosemide is a drug often used in the treatment of nephrotic syndrome. The study conducted by Keller et al. is focused on evaluating the pharmacokinetics of furosemide in patients with nephrotic syndrome and it showed the diminished protein binding of furosemide from 98.6% to 97.2% due to low albumin levels in nephrotic patients that were administered 40 mg of furosemide intravenously. The AUC was 166 ± 36 and 237 ± 46 μg min/mL for nephrotic patients and control group consecutively. The mean free fraction of furosemide was elevated in patients with nephrotic syndrome (0.028 vs. 0.014). The volume of urine, sodium excretion and furosemide excretion were significantly decreased in nephrotic patients (P < 0.001, P < 0.005, P < 0.005) which suggest worse response to treatment. Nevertheless, the reduced response to the diuretic in those patients is more likely, according to the authors, the result of renal impairment rather than the low albumin levels themselves. The research also showed the increased non-renal clearance of furosemide in patients with nephrotic syndrome (Sjöström et al., 1989). In the study conducted by Sjöström et al. administration of dextran increased plasma volume, which lowered the albumin concentrations and consecutively increased the volume of distribution and renal and non-renal clearances of furosemide. They decreased after the albumin infusion, which suggests the correlation of lower serum albumin concentrations with higher clearances (Keller et al., 1982). Some studies indicate that reduced albumin levels can lead to unsatisfactory transportation of the diuretics to kidneys but the correlation between hypoalbuminemia and diuretic resistance remains uncertain (Sjöström et al., 1989; Elwell et al., 2003).

Klinkmann et al. focused on not only albumin concentration but also their function expressed in albumin binding capacity (ABiC). The study showed a correlation between ABiC and glomerular filtration rate (GFR) (P = 0.003), describing decreased ABiC accompanying renal impairment. It also correlates with a free furosemide fraction that is significantly higher in patients with ABiC ≤60% than in those with ABiC ≥60% (P < 0.001). They also observed a positive correlation between ABiC and the response to initiation of furosemide described as urine output to fluid intake ratio. ABiC could potentially serve as a marker to predict diuretic response, but further research is necessary to confirm its effectiveness (Klinkmann et al., 2022).

The simultaneous use of furosemide and albumin is a possible treatment option. The meta-analysis of various studies on this topic has suggested that this form of treatment may only be advantageous for specific groups of patients, such as those with a basal albumin concentration that is below 2.5 g/dL, those with impaired renal function, or those who receive >30 mg of albumin (Lee et al., 2021).

The urinary excretion of furosemide and diuretic resistance in hypoalbuminemic patients with heart failure is not associated with their plasma albumin concentration (Lee et al., 2021; Charokopos et al., 2019). Moreover, no correlation between albumin levels and the urinary excretion of furosemide was found in the study on critically ill hypoalbuminemic patients with cardiovascular and cerebral disorders, infections, malignancies, traumas. The simultaneous administration of furosemide with albumin was also not beneficial for those patients in the six and 8-h follow-up (Mahmoodpoor et al., 2020).

#### 3.7.2 Vitamin D

A biologically active metabolite of vitamin D is 1,25-dihydroxyvitamin D (1,25(OH)2D), which has various metabolic effects. Its crucial function is to preserve calcium homeostasis. The initial hydroxylation of vitamin D takes place in the liver, resulting in the production of 25-hydroxyvitamin D (25(OH)D). The subsequent hydroxylation occurs in the kidneys, leading to the formation of 1,25-dihydroxyvitamin D. Studies suggest vitamin D protein binding above 99%. The majority of it binds to vitamin D binding globulin (DBP), but it also binds to albumin (Bikle and Schwartz, 2019).

The study conducted by van Hoof et al. showed lower total and free 1,25(OH)2D and 25(OH)D serum concentrations in patients with nephrotic syndrome compared to controls. Surprisingly, a positive correlation between total and free 1,25(OH)2D and albumin concentration was proven, but not with DBP. Nephrotic patients presented hypoalbuminemia, meanwhile, their DBP levels were within normal range (Traeger and Haug, 1986). This may indicate that the loss of albumin-bound 1,25(OH)2D and 25(OH)D in the urine is more significant than the DBP-bound fraction in those patients, even though DBP binds more than 85% of vitamin D. Other potential explanations for reduced total and free 1,25(OH)2D concentrations include lower steady state and incapability of 1a-hydroxylase activity due to impairment of tubular function (van Hoof et al., 2001). Furthermore, this may contribute to enhanced bone resorption and various bone disorders in individuals with nephrotic syndrome (Malluche et al., 1979). As a contradiction, data in the literature also present nephrotic patients with significantly lowered DBP levels compared to a control group, yet still mostly within the normal range (Barragry et al., 1977; Schmidt-Gayk et al., 1977).

Studies carried out on individuals with hypoalbuminemia caused by ailments other than nephrotic syndrome have also exhibited a link between low levels of plasma albumin and a deficiency of vitamin D (Chiang et al., 2021; Lai et al., 2015; Bhan et al., 2010). In a study conducted by Lai et al., patients with cirrhosis were compared based on their plasma albumin levels. The study found that both total 25(OH)D and free 25(OH)D were significantly lower in patients with low albumin levels compared to those with normal albumin levels. However, the percentage of free 25(OH)D was significantly higher in patients with low al-bumin levels. Additionally, patients with hypoalbuminemia also had lower 1,25(OH)2D (Bhan et al., 2010). The cross-sectional study performed on older adults with hip fracture identified low albumin levels as the only significant risk factor of vitamin D deficiency among the other potential risk factors examined (Chiang et al., 2021). In the study on dialysis patients, the majority had a 25(OH)D deficiency, which was associated with low plasma albumin. This may indicate the need to consider empiric therapy with the nutritional form of vitamin D for these patients, especially during the winter season (Bhan et al., 2010).

#### 3.7.3 Lovastatin

Lovastatin is an HMG - CoA reductase inhibitor, used to reduce elevated levels of low-density lipoprotein cholesterol (LDL). It is mainly used as the primary and secondary prevention of cardiovascular disease. More than 95% of lovastatin and its β-hydroxy acid metabolite bind to human plasma proteins. Lovastatin’s use in nephrotic syndrome or proteinuria above 3 g/day is to reduce coexisting hyperlipidemia. It is effective in lowering serum total cholesterol, LDL and apolipoprotein B (Chan et al., 1992; Vega and Grundy, 1988). In the study conducted by Chan et al., the efficacy of lovastatin was confirmed, no significant changes in proteinuria, serum albumin and renal albumin clearance were observed. The treatment should be under control as adverse effects such as drug-dependent rhabdomyolysis and severe hypertriglyceridemia appeared during the study (Chan et al., 1992).

#### 3.7.4 Dexmedetomidine

Dexmedetomidine is a highly selective alpha-2 agonist used for sedation. In healthy individuals, around 94% of plasma dexmedetomidine is bound to albumin. Iirola et al. reported a study of dexmedetomidine pharmacokinetics and pharmacodynamics during a long-term infusion (>48 h) in patients of an intensive care unit. The dosages were selected individually for each patient to achieve an optimal sedation. The decrease in albumin concentration was found to result in a significant increase in the distribution volume at steady state (VdSS). The study also demonstrated that the elimination half-life of dexmedetomidine was prolonged in hypoalbuminemic patients (Iirola et al., 2012). Nevertheless, in another study, Zhang et al. presented a shortened distribution half-life (3.2 ± 2.2 vs. 5.0 ± 3.6 min, P < 0001) and the diminished maximum concentration of dexmedetomidine (Cmax = 0.52 ± 0.10 vs. 0.66 ± 0.13 μg/mL, P < 0.01) in nine patients with mean plasma albumin levels of 28.6 ± 3.6 g/L compared to patients with normoalbuminemia. They also confirmed the previously stated significant increase in VdSS in those patients (7.18 ± 1.63 vs. 5.11 ± 1.12 L/kg, P < 0.001). The drug’s efficacy did not differ significantly between the groups in terms of blood pressure, heart rate and sedation effect measured by Ramsay sedation score. No serious adverse reactions were noted in any of the patients (Zhang et al., 2015).

#### 3.7.5 Theophylline

Theophylline is a xanthine used in pulmonary diseases such as asthma or chronic obstructive pulmonary disease. It acts by relaxing smooth muscles and suppressing the reaction of the respiratory passages to external influences. Approximately 40% binds with proteins, mainly albumin. Nakura et al. conducted a study on rats that were given a single injection of 10 mg/kg of theophylline. The study reported that rats with hypoalbuminemia had an increased total clearance of theophylline. Furthermore, the concentration of unbound theophylline was almost identical to the total concentration and the volume of distribution significantly increased (Nakura et al., 1998). The research conducted on patients with hypoalbuminemia showed a decreased total concentration of theophylline in blood in comparison to patients with normal albumin levels. However, hypoalbuminemic patients had a higher unbound fraction (Zarowitz et al., 1985).

#### 3.7.6 Warfarin

Warfarin is one of the most commonly used anticoagulants. It inhibits vitamin K, which prevents the activation of vitamin K - dependent coagulation factors. Approximately 99% of warfarin is bound with proteins in the blood, mainly with albumin. Piroli et al. presented the study comparing warfarin’s pharmacokinetics in a woman with idiopathic hypoalbuminemia with a group of healthy individuals. They found statistically significant differences: increased clearance and reduced half-life of plasma warfarin in the hypoalbuminemic patient. Also, the unbound fraction of warfarin was elevated (Dautzenberg et al., 2018). Other studies presented the correlation between lower albumin plasma concentration and higher incidence of international normalized ratio of prothrombin time (PT-INR) >3.0 and major bleeding events in patients with atrial fibrillation treated with warfarin (Kawai et al., 2019; Tincani et al., 2002). Thus, albumin may serve as a useful marker for monitoring the administration of warfarin and preventing supratherapeutic PT-INR levels, successively reducing the likelihood of adverse effects associated with the use of warfarin (Kawai et al., 2019; Fender and Dobrev, 2019).

#### 3.7.7 Donepezil

Donepezil is a selective and reversible acetylcholinesterase inhibitor used in Alzheimer’s disease. Donepezil binds to protein molecules by 96%, and a significant pro-portion of this binding involves albumin, with approximately 75% of the drug binding to this protein. Patients with varying levels of albumin were studied, and it was found that those with low albumin levels showed a more positive response to treatment than those with high levels of albumin. Further research is required to determine the relationship between albumin concentration and treatment efficacy. However, it is im-portant to consider this factor while evaluating the topic (Rozzini et al., 2008).

#### 3.7.8 Oncological drugs

Cancer patients are particularly vulnerable to hypoalbuminemia, a condition precipitated by various factors such as the increased release of proinflammatory cytokines and growth factors, which suppress albumin synthesis, alongside increased vascular permeability and deteriorated nutritional status. Despite the prevalence of hypoalbuminemia in oncology, there is a notable paucity of research examining its impact on pharmacokinetics and the free drug levels in this patient population. One of available studies was performed by Murdock et al. who retrospectively assessed the tolerability of oral anticancer therapies in hypoalbuminemic patients (Murdock et al., 2021). Many oncological agents, particularly tyrosine kinase inhibitors (TKIs), exhibit high protein-binding affinity, suggesting a potential for increased toxicity due to reduced plasma protein availability. Contrary to expectations, described study find no significant difference in the incidence of adverse effects between normoalbuminemic and hypoalbuminemic patients treated with a targeted oral oncolytic drug exhibiting >95% protein binding. However, hypoalbuminemic patients experienced a markedly higher rate of therapy discontinuation due to documented toxicities, potentially attributable to either more severe adverse effects or a compromised functional status in this cohort. Another study focusing on TKIs performed by J.Marini et al. examined larger group of patients – 220 – and their findings support those of Murdock’s–presence of hypoalbuminemia was associated with earlier and higher rate of TKIs discontinuation in comparison with normoalbuminemic patients (Marini et al., 2022). Other group of oncological drugs that gained great recognition in the treatment of particular cancer types are anti-programmed cell death receptor-1 monoclonal antibodies such as nivolumab. Findings from the study, assessing clinical and pharmacological parameters influencing nivolumab toxicity, performed by L. Hirsch et al. proved that hypoalbuminemia is associated both with–low nivolumab Cmin on day 14 – and increased risk of immune-related acute limiting toxicity (irALT) (Hirsch et al., 2020). The study however found no association between plasma Cmin and toxicity, what suggest that increased risk of irALT is not related to increased nivolumab plasma exposure. The principal limitation of the studies cited lies in their failure to measure free drug concentrations and assess pharmacokinetic alterations, which are critical for evaluating the safety and efficacy of oncological therapies in hypoalbuminemic states.

A related study by Arrieta et al. highlighted the heightened toxicity of intravenous chemotherapeutic agents, such as cisplatin (with >90% protein binding) and paclitaxel (89%–98% protein binding), in hypoalbuminemic patients with non-small cell lung cancer (NSCLC), compared to their normoalbuminemic counterparts (Arrieta et al., 2010). Findings from different study confirm that initial low albumin level before chemotherapy can affect plasma exposure of paclitaxel, so it is recommended to determine albumin level before drug administration to reduce occurrence and severity of adverse effects, such as myelosuppression (Fan et al., 2022). In hypoalbuminemia there is a higher unbound proportion of chemotherapeutic drugs and increased time of drug’s clearance causing delayed elimination–that may be reasons why patients with lower level of albumin developed more adverse reactions (Manasa et al., 2023). Binding to albumin is important for increasing drug concentration at the tumor target and improving their *in vivo* therapeutic efficacy, that is why albumin can stand as a promising biomaterial for chemotherapeutic drug delivery. Potentially, drugs can be synthesized as nanoparticles in conjugation with albumin, thus allowing more efficient delivery to the tumor site and prolonged blood circulation (Yu et al., 2022). Another solution to presented problem may be adding albumin to the treatment regimen, as it appears to be able to increase the binding of drug to albumin, therefore to increase half-life of circulating drug and its entry into cancer cells, making it useful way to modify treatment (Bagheri and Saboury, 2021).

##### 3.7.8.1 Tyrosine kinase inhibitors

Regorafenib is an oral small-molecule tyrosine kinase inhibitor targeting vascular endothelial growth factor receptor, fibroblast growth factor receptor and platelet-derived growth factor receptor. It is approved for treatment of hepatocellular carcinoma (HCC), gastrointestinal stromal tumors (GIST) and colorectal cancer (CRC). In the study performed in 2024 the total concentration of regorafenib and its metabolites in hypoalbuminemic patients was determined to be below target plasma concentration ranges, what may contributed to shorter overall survival in the hypoalbuminemic group of patients. Possible explanation of this outcome is poorer nutritional status in this group and lower doses of regorafenib received. Monitoring concentration of regorafenib regularly is necessary to guide dose adjustment, however there is necessity for more research including measurement of free regorafenib concentration (Fujita et al., 2024).

Afatinib is a TKI targeting epidermal growth factor receptor (EGFR) and is used in the treatment of NSCLC. Study performed by T. Takahashi et al. evaluated relationship between afatinib plasma concentration and occurrence of adverse effects and proved that higher plasma concentration of afatinib induced adverse effects, so monitoring it may be useful in predicting side effects and improve patients’ quality of life Serum albumin level was however the only laboratory data showing a correlation with the plasma concentration of afatinib and it may offer an indicator of elevated exposure to afatinib. These findings are promising but further research targeting the relationship between serum afatinib concentration, and serum albumin is essential to predict possible dose reduction (Takahashi et al., 2021).

Pazopanib is an angiogenesis inhibitor targeting vascular endothelial growth factor receptor, platelet-derived growth factor receptor and c-KIT. Drug is approved for treatment of renal cell carcinoma and sarcoma. H. Tanaka et al. performed study to assess possibility of drug’s dose reduction to decrease occurrence of adverse effects. The main finding is that pazopanib dose is not significantly associated with pazopanib concentration, contrary to the level of albumin - dose of pazopanib can be reduced from 800mg to 400 mg (while maintaining the Cmin) if albumin level is within normal range (Tanaka et al., 2020). Different study assessed impact of albumin level on free pazopanib concentration and proved that lower albumin level indicate higher unbound fraction of the drug. However there were no statistically significant differences between free drug concentration in the range of plasma albumin levels of 29–44 g/L. The effect of varying albumin levels on unbound fraction of pazopanib in patient plasma would need to be further investigated using a larger sample size with wider albumin range for it to be meaningful (Toh et al., 2020).

Imatinib is a small-molecule TKI targeting BCR-ABL fusion protein, KIT and PDGFR that was first developed for the treatment of chronic myeloid leukemia (CML), but currently is also used in the treatment of GISTs. Study performed by H Xu et al. proved that patients with higher blood imatinib concentrations are more likely to develop thrombocytopenia and higher protein levels in the body can significantly reduce the concentrations of free imatinib, affecting their pharmacological effects (Xu and Liu, 2021). More research assessing pharmacokinetics of imatinib is needed, but this study indicates that protein level can be used to predict occurrence of serious adverse effects.

#### 3.7.9 Albuminemia in the design of long-acting drugs

Various ligands binding to albumin have been identified to use albumin as a carrier for drugs–such as small molecules, peptides, proteins. Some of them–domain antibodies or small molecules–extended the half-life of substrates otherwise rapidly cleared by renal filtration. Using fatty acids as albumin tags was also investigated, resulting in approval of Insulin detemir–being the first protein modified by fatty acid. Insulin degludec is the latest generation of albumin binding insulin modified by fatty acids. Increasing half-life of insulin is possible due to primarily subcutaneous application, with a small increase in intravenous half-life because of albumin binding. (Knudsen and Lau, 2019).

Glucagon-like peptide-1 (GLP-1) is a 30 amino acid peptide hormone with a short halflife - 1.5 min following intravenous dosing and 1.5 h following subcutaneous dosing in humans. Semaglutide, a GLP-1 receptor agonist, is primarily utilized in the management of type 2 diabetes. In addition to its glycemic control properties, clinical trials have demonstrated its efficacy in reducing glycated hemoglobin levels and promoting weight loss. Semaglutide exhibits high plasma protein binding, with approximately 99% bound to albumin. This extensive protein binding significantly contributes to its prolonged half-life of approximately 1 week, allowing for convenient once-weekly dosing regimens (Tan et al., 2021). A study conducted by Jensen et al. investigated the pharmacokinetic profile of semaglutide in patients with hypoalbuminemia secondary to hepatic impairment (Jensen et al., 2018). The findings indicated that the maximum concentration (Cmax) of semaglutide was not significantly affected by hepatic impairment or reduced albumin levels. These results suggest that dosage adjustments for semaglutide may not be necessary in patients with hepatic impairment. Liraglutide–another GLP-1 agonist is administered once daily. It was achieved by preparing analogs that could bind to albumin in reversible manner and protect peptide from degradation and renal filtration. Use of palmitate with a γGlu linker was optimal to achieve an appropriate *in vivo* protraction without compromising receptor potency (Knudsen and Lau, 2019).

## 4 Conclusion

Adjustment of drug dosage is necessary especially in conditions such as: nephrotic syndrome, cirrhosis or sepsis where hypoalbuminemia is prevalent. The results of studies emphasize the need for individualized dosing regimens based on TDM to optimize drug therapy, avoiding subtherapeutic or toxic drug levels in patients with hypoalbuminemia. The review outlines changes in the pharmacokinetic parameters observed in hypoalbuminemia that are summarized in Table 1 which can be treated as a guide for clinicians. There are, however several limitations of the review including: reliance on the studies with small sample sizes, little amount of studies measuring free drug concentration and lack of data on the impact of hypoalbuminemia of many drugs. There is a need to conduct future research focusing on measurement of free drug concentrations–especially of novel therapeutic agents, such as biologic treatment or immunotherapies, and to understand long-term effects of hypoalbuminemia and altered pharmacokinetics on patient outcomes.

**TABLE 1:** Changes in pharmacokinetic parameters in hypoalbuminemia.

Drug	Study group size	Albumin (g/dL)	Protein binding (%)	Effect of hypoalbuminemia (treatment ineffectiveness/treatment toxicity/no changes)	Changes in PK/PD
Penicillin G	46	2.6	45%–68%	-	↑C_max_
Flucloxacillin	2	1.65	93%	failure to achieve therapeutic effect/flucloxacillin toxicity	↑C_max_/↓C_max_
Ceftriaxone	36 8 (animal)	2.39 2.61	95%	failure to achieve therapeutic effect -	↓C_max_ ↓AUC_0–24_, ↑CL, Vd↑
Ertapenem	8 (animal)	2.61	90%	-	↓AUC_0–24_, ↑CL
Gentamicin	66 + 50 1,049/260	2.5/2.9 2.8/3.2	15%	-subtherapeutic drug concentration	↓C_max_ Vd↑
Tobramycin	1,049/260	2.8/3.2	30%	subtherapeutic drug concentration	Vd↑
Vancomycin	67	3.21	50%	subtherapeutic drug concentration	↓C_max_,↑CL, Vd↑
Teicoplanin	13	2.8	90%	subtherapeutic drug concentration	↑C_free_
Prednisolone	7	2.44	65%–91%	-	↓C_max_,↑CL, VDss↑ ↓AUC_0–24_ ↑C_free_ ↓AUC_free(0–24)_
Cyclosporine	19	2.13	90% (mainly lipoproteins)	no correlation	-
Mycophenolate mofetil	23 51 20 1	2.65–4.55 - 2.5–3.5 2.1	97%	Subtherapeutic AUC level, total MPA concentration may not increase in the presence of severe hypoalbuminemia	↑C_free_, ↓AUC_0–24_
Methotrexate	32	<3.5	50%	MTX third spacing and accumulation	↑CL
Naproxen	1 6	2.5 2.7–3.5	>95%	efficacy of Rheumatoid Arthritis naproxen treatment may be even improved	↓C_max_, ↑C_free_, ↑Vd, ↑CL, ↓CL_free_
Itraconazole	1	2.8	99,80%	-	↑C_max_, ↑AUC_0–24_
Posaconazole	1	2.5	>98%	failure to achieve therapeutic effect	-
Voriconazole	67 1	3.4 2.5	58%	potentionally toxic trough concentration patients full recovery	↑C_min_
Phenytoin	1 1	0.2 2.3	90%	phenytoin toxicity-lethargy, bilateral clonus and tremors phenytoin toxicity- ataxia and drowsiness	↑C_max_,↑C_free_ ↑C_free_
Valproic Acid	1 15 1	1.4 >4 2.68	80%–95%	valproic acid toxicity- cognitive impairment valproic acid toxicity- hyperaammonemia, elevated transaminanese, thrombocytopenia valproic acid toxicity- difficulty walking, involuntary muscle jerks	↑C_free_ ↑C_free_ ↑C_max_,↑C_free_
Furosemide	-	-	91%–99%	uncertain contribution to diuretic resistance in nephrotic syndrome	↑CL, ↑C_free_, ↓AUC_0–24_
Vitamin D	-	-	>99%	bioactive vitamin D deficiency	↓C, ↓C_free_
Dexmedetomidine	21 9	1.35 <3.5	94%	no alterations in efficacy and adverse effects	↑ VDss, ↓/↑ t½, ↓C_max_
Theophylline	- (animal) 9	- 2.7 +- 0.61	40%	-	↑Vd, ↑ CL↓ C_max_, ↑ C_free_
Warfarin	1 28 65	not specified hypoalbuminemia <3.6 3.17 +- 0.24	99%	higher incidence of over-anticoagulation	↑ CL, ↓t½, ↑ C_free_
Donepezil	37	≤4	75%	better response to treatment	-
Lovastatin	-	-	95%	risk of drug toxicity	-
Diazepam	1	2.7	98%–99%	coma	increased desmethyldiazepam concentration, diazepam concentration within norm
Phenobarbital	1	2.7	20%–45%	coma	phenobarbital concentration within norm, free fraction not measured
Regorafenib	21	-	99%	Treatment ineffectiveness	↓C_max_
Afatinib	24	-	95%	Treatment toxicity	↑C_free_
Pazopanib	43	-	99%	-	↑C_free_
Imatinib	43	-	95%	Treatment toxicity	↑C_free_
Semaglutide	44	-	99%	No changes	-

## Glossary

ABiC: albumin binding capacity
ARC: augmented cretinine clearance
AIDS: acute immunodeficiency syndrome
AUC: are under curve
CL: clearance
Cmax: peak concentration
Cmin: minimum concentration
CML: chronic myeloid leukemia
COX: cyclooxygenase
CRC: colorectal cancer
CsA: cyclosporine A
CSF: cerebrospinal fluid
DBP: vitamin D binding globulin
eCLcr: estimated creatinine clearance
EGFR: epidermal growth factor receptor
ER: extraction ratio
fT > MIC: percent of time free drug concentration remains above the minimum inhibitory concentration
GIST: gastro-intestinal stromal tumor
GLP-1: glucagon like peptide 1
HCC: hepatocellular carcinoma
HDL: high density lipoprotein
HIV: human immunodeficiency virus
HMG-CoA: hydroxy methylo-glutarylo coenzyme A
IE: infective endocarditis
IMPDH: inosine monophosphate dehydrogenase
irALT: immune related acute limiting toxicity
ITZ: itraconazole
LDL: low density lipoprotein cholesterol
MIC: minimum inhibitory concentration
MMF: mycophenolate mofetil
MPA: mycophenolate acid
MRSA: meticilin resistant *staphylococcus aureus*
MTX: methotrexate
NR: normal range
NS: nephrotic syndrome
NSAIDs: non steroidal anti-inflammatory drugs
NSCLC: non-small cell lung cancer
PAE: post antibiotic effect
PHT: phenytoin
PT: prothrombin time
PTA: probability of target attainment
RRT: renal replacement therapy
SAP: severe acute pancreatitis
TDM: therapeutic drug monitoring
TKI: tyrosine kinase inhibitor
tVPA: total valproic acid serum concentration
Vd: volume distribution
VDss: the steady state volume of distribution
VPA: valproic acid

## References

[B1] Abdel-Mottaleb M. M. A. Boi L. Barra M. Colin J. Berni L. Béduneau A. (2023). Lipoproteins as drug carriers for cyclosporine A: optimization of the entrapment. Mater. (Basel) 16 (3), 1156. 10.3390/ma16031156 PMC9918909 36770166

[B2] Ackland S. P. Schilsky R. L. (1987). High-dose methotrexate: a critical reappraisal. J. Clin. Oncol. 5, 2017–2031. 10.1200/JCO.1987.5.12.2017 3316519

[B3] Alex R. Mathew M. Arul S. Kundavaram A. (2014). Overdose of mycophenolate mofetil managed in a secondary care hospital in South India. Indian J. Pharmacol. 46 (3), 337–338. 10.4103/0253-7613.132191 24987184 PMC4071714

[B4] Allison A. C. (2005). Mechanisms of action of mycophenolate mofetil. Lupus. 14 (Suppl. 1), s2–s8. 10.1191/0961203305lu2109oa 15803924

[B5] Arrieta O. Michel Ortega R. M. Villanueva-Rodríguez G. Serna-Thomé M. G. Flores-Estrada D. Diaz-Romero C. (2010). Association of nutritional status and serum albumin levels with development of toxicity in patients with advanced non-small cell lung cancer treated with paclitaxel-cisplatin chemotherapy: a prospective study. BMC cancer 10, 50. 10.1186/1471-2407-10-50 20170547 PMC2843671

[B6] Bagheri S. Saboury A. A. (2021). Hypothesis of using albumin to improve drug efficacy in cancers accompanied by hypoalbuminemia. Xenobiotica 51 (7), 778–785. 10.1080/00498254.2021.1929557 33979263

[B7] Bailey D. N. Briggs J. R. (2004). Gentamicin and tobramycin binding to human serum *in vitro* . J. Anal. Toxicol. 28 (3), 187–189. 10.1093/jat/28.3.187 15107149

[B8] Bailey E. M. Rybak M. J. Kaatz G. W. (1991). Comparative effect of protein binding on the killing activities of teicoplanin and van-comycin. Antimicrob. Agents Chemother. 35 (6), 1089–1092. 10.1128/AAC.35.6.1089 1834010 PMC284292

[B9] Ballmer P. E. (2001). Causes and mechanisms of hypoalbuminaemia. Clin. Nutr. 20 (3), 271–273. 10.1054/clnu.2001.0439 11407876

[B10] Barakat S. Assem H. Salama M. Mikhael N. El Chazli Y. (2022). Is hypoalbuminemia a risk factor for high-dose methotrexate toxicity in children with acute lymphoblastic leukemia? J. Egypt Natl. Canc Inst. 34 (1), 17. 10.1186/s43046-022-00122-7 35434757 PMC13314256

[B11] Barbarino J. M. Staatz C. E. Venkataramanan R. Klein T. E. Altman R. B. (2013). PharmGKB summary: cyclosporine and tacrolimus pathways. Pharmacogenet Genomics 23 (10), 563–585. 10.1097/FPC.0b013e328364db84 23922006 PMC4119065

[B12] Barragry J. M. France M. W. Carter N. D. Auton J. A. Beer M. Boucher B. J. (1977). Vitamin-D metabolism in nephrotic syndrome. Lancet 2 (8039), 629–632. 10.1016/s0140-6736(77)92498-9 71448

[B13] Bergrem H. (1983). Pharmacokinetics and protein binding of prednisolone in patients with nephrotic syndrome and patients undergoing hemodialysis. Kidney Int. 23 (6), 876–881. 10.1038/ki.1983.110 6887699

[B14] Bhan I. Burnett-Bowie S. A. Ye J. Tonelli M. Thadhani R. (2010). Clinical measures identify vitamin D deficiency in dialysis. Clin. J. Am. Soc. Nephrol. 5 (3), 460–467. 10.2215/CJN.06440909 20185603 PMC2827576

[B15] Bikle D. D. Schwartz J. (2019). Vitamin D binding protein, total and free vitamin D levels in different physiological and pathophysiological conditions. Front. Endocrinol. (Lausanne) 10, 317. 10.3389/fendo.2019.00317 31191450 PMC6546814

[B16] Boodhan S. Friedman J. N. (2009). Naproxen-induced anemia and hypoalbuminemia in a 16-month-old child. Clin. Pediatr. (Phila). 48 (1), 96–98. 10.1177/0009922808321900 18632553

[B17] Bukkems L. H. Roger C. Hodiamont C. J. Lefrant J. Y. Juffermans N. P. Roberts J. A. (2018). Predictive performance of a gentamicin population pharmacokinetic model in two western populations of critically ill patients. Int. J. Antimicrob. Agents 52 (2), 218–225. 10.1016/j.ijantimicag.2018.04.016 29704547

[B18] Bullingham R. E. Nicholls A. J. Kamm B. R. (1998). Clinical pharmacokinetics of mycophenolate mofetil. Clin. Pharmacokinet. 34, 429–455. 10.2165/00003088-199834060-00002 9646007

[B19] Burger D. M. Meenhorst P. L. Mulder J. W. Kraaijeveld C. L. Koks C. H. Bult A. (1994). Therapeutic drug monitoring of phenytoin in patients with the acquired immunodeficiency syndrome: therapeutic drug. Monitoring 16 (6), 616–620. 10.1097/00007691-199412000-00015 7878704

[B20] Butterfield J. M. Patel N. Pai M. P. Rosano T. G. Drusano G. L. Lodise T. P. (2011). Refining vancomycin protein binding estimates: identification of clinical factors that influence protein binding. Antimicrob. Agents Chemother. 55 (9), 4277–4282. 10.1128/AAC.01674-10 21670191 PMC3165330

[B21] Cabrerizo S. Cuadras D. Gomez-Busto F. Artaza-Artabe I. Marín-Ciancas F. Malafarina V. (2015). Serum albumin and health in older people: review and meta analysis. Maturitas 81 (1), 17–27. 10.1016/j.maturitas.2015.02.009 25782627

[B22] Challis J. R. Lockwood C. J. Myatt L. Norman J. E. Strauss J. F. 3rd Petraglia F. (2009). Inflammation and pregnancy. Reprod. Sci. 16 (2), 206–215. 10.1177/1933719108329095 19208789

[B23] Chan P. C. Robinson J. D. Yeung W. C. Cheng I. K. Yeung H. W. Tsang M. T. (1992). Lovastatin in glomerulonephritis patients with hyperlipidaemia and heavy proteinuria. Nephrol. Dial. Transpl. 7 (2), 93–99. 10.1093/oxfordjournals.ndt.a092102 1314986

[B24] Charokopos A. Griffin M. Rao V. S. Inker L. Sury K. Asher J (2019). Serum and urine albumin and response to loop diuretics in heart failure. Clin. J. Am. Soc. Nephrol. 14 (5), 712–718. 10.2215/CJN.11600918 31010938 PMC6500945

[B25] Chiang M. H. Kuo Y. J. Chang W. C. Wu Y. Lin Y. C. Jang Y. C. (2021). Association of vitamin D deficiency with low serum albumin in Taiwanese older adults with hip fracture: a prospective cross-sectional study. J. Nutr. Sci. Vitaminol. (Tokyo) 67 (3), 153–162. 10.3177/jnsv.67.153 34193674

[B26] Curran M. Simpson D. Perry C. (2003). Ertapenem: a review of its use in the management of bacterial infections. Drugs 63 (17), 1855–1878. 10.2165/00003495-200363170-00006 12921489

[B27] Dautzenberg G. Nederlof M. Beekman A. Egberts T. Heerdink E. R. (2018). Severe cognitive impairment associated with a high free but therapeutic total concentration of valproic acid due to hypoalbuminemia in an older patient with bipolar disorder. J. Clin. Psychopharmacol. 38 (3), 265–268. 10.1097/JCP.0000000000000872 29621091

[B28] De Beule K. Van Gestel J. (2001). Pharmacology of itraconazole. Drugs 61 (Suppl. 1), 27–37. 10.2165/00003495-200161001-00003 11219548

[B29] Deitchman A. N. Singh R. S. P. Derendorf H. (2018). Nonlinear protein binding: not what you think. J. Pharm. Sci. 107 (7), 1754–1760. 10.1016/j.xphs.2018.03.023 29626534 PMC6042850

[B30] Dhanani J. A. Ahern B. Lupinsky L. Jackson K. Wallis S. C. Abdul-Aziz M. H. (2020). Comparative plasma pharmacokinetics of ceftriaxone and ertapenem in normoalbuminemia, hypoalbuminemia, and albumin replacement in a sheep model. Antimicrob. Agents Chemother. 64 (7), 025844-19–e2619. 10.1128/AAC.02584-19 PMC7318000 32366707

[B31] Elwell R. J. Spencer A. P. Eisele G. (2003). Combined furosemide and human albumin treatment for diuretic-resistant edema. Ann. Pharmacother. 37 (5), 695–700. 10.1345/aph.1C320 12708949

[B32] Etzel J. V. Nafziger A. N. Bertino J. S. Jr (1992). Variation in the pharmacokinetics of gentamicin and tobramycin in patients with pleural effusions and hypoalbuminemia. Antimicrob. Agents Chemother. 36 (3), 679–681. 10.1128/AAC.36.3.679 1622185 PMC190580

[B33] Fan W. Yin W. Zhou F. Wang Y. Fan J. Zang F. (2022). The correlation between paclitaxel chemotoxicity and the plasma albumin level in cancer patients. J. Clin. Pharm. Ther. 47 (12), 2237–2244. 10.1111/jcpt.13798 36325658

[B34] Fanali G. di Masi A. Trezza V. Marino M. Fasano M. Ascenzi P. (2012). Human serum albumin: from bench to bedside. Mol. Aspects Med. 33 (3), 209–290. 10.1016/j.mam.2011.12.002 22230555

[B35] Faulds D. Goa K. L. Benfield P. (1993). Cyclosporin. A review of its pharmacodynamic and pharmacokinetic properties, and therapeutic use in immunoregulatory disorders. Drugs 45 (6), 953–1040. 10.2165/00003495-199345060-00007 7691501

[B36] Fender A. C. Dobrev D. (2019). Bound to bleed: how altered albumin binding may dictate warfarin treatment outcome. Int. J. Cardiol. Heart Vasc. 22, 214–215. 10.1016/j.ijcha.2019.02.007 30963099 PMC6437285

[B37] Ferry J. J. Horvath A. M. Bekersky I. Heath E. C. Ryan C. F. Colburn W. A. (1988). Relative and absolute bioavailability of prednisone and prednisolone after separate oral and intravenous doses. J. Clin. Pharmacol. 28 (1), 81–87. 10.1002/j.1552-4604.1988.tb03105.x 3350994

[B38] Forsythe P. Paterson S. (2014). Ciclosporin 10 years on: indications and efficacy. Vet. Rec. 174 (Suppl. 2), 13–21. 10.1136/vr.102484 24682697 PMC3995292

[B39] Franch-Arcas G. (2001). The meaning of hypoalbuminaemia in clinical practice. Clin. Nutr. 20 (3), 265–269. 10.1054/clnu.2001.0438 11407875

[B40] Freeman D. J. (1991). Pharmacology and pharmacokinetics of cyclosporine. Clin. Biochem. 24 (1), 9–14. 10.1016/0009-9120(91)90084-r 2060139

[B41] Fujita K. Taguchi D. Fukuda K. Yoshida T. Shimazu K. Shinozaki H. (2024). Association between albumin-bilirubin grade and plasma trough concentrations of regorafenib and its metabolites M-2 and M-5 at steady-state in Japanese patients. Investig. new drugs 42 (3), 252–260. 10.1007/s10637-024-01429-z 38517650 PMC11164767

[B42] Fulton J. D. McGovern E. M. McCarron B. N. Carlile A. K. (1988). Phenytoin monitoring: clinical or scientific? Scott Med. J. 33 (3), 267–268. 10.1177/003693308803300305 3175605

[B43] Gidal B. E. Collins D. M. Beinlich B. R. (1993). Apparent valproic acid neurotoxicity in a hypoalbuminemic patient. Ann. Pharma-cother 27 (1), 32–35. 10.1177/106002809302700107 8381686

[B44] Guilhaumou R. Benaboud S. Bennis Y. Dahyot-Fizelier C. Dailly E. Gandia P. (2019). Optimization of the treatment with beta-lactam antibiotics in critically ill pa-tients-guidelines from the French Society of Pharmacology and Therapeutics (Société Française de Pharmacologie et Thérapeutique-SFPT) and the French Society of Anaesthesia and Intensive Care Medicine (Société Française d'Anesthésie et Réanimation-SFAR). Crit. Care 23 (1), 104. 10.1186/s13054-019-2378-9 30925922 PMC6441232

[B45] Hartman S. J. F. Upadhyay P. J. Hagedoorn N. N. Mathôt R. A. A. Moll H. A. van der Flier M. (2021). Current ceftriaxone dose recommendations are adequate for most critically ill children: results of a population pharmacokinetic modeling and simulation study. Clin. Pharmacokinet. 60 (10), 1361–1372. 10.1007/s40262-021-01035-9 34036552 PMC8505376

[B46] Hayashi Y. Lipman J. Udy A. A. Ng M. McWhinney B. Ungerer J. (2013). β-Lactam therapeutic drug monitoring in the critically ill: optimising drug exposure in patients with fluctuating renal function and hypoalbuminaemia. Int. J. Antimicrob. Agents 41 (2), 162–166. 10.1016/j.ijantimicag.2012.10.002 23153962

[B47] He J. Mao E. Q. Feng J. Jiang H. T. Yang W. H. Chen E. Z. (2016). The pharmacokinetics of vancomycin in patients with severe acute pancreatitis. Eur. J. Clin. Pharmacol. 72 (6), 697–702. 10.1007/s00228-016-2018-0 26902230

[B48] Heffernan A. J. Sime F. B. Kumta N. Wallis S. C. McWhinney B. Ungerer J. (2022). Multi-center population pharmacokinetic study of unbound ceftriaxone in critically ill patients. Antimicrob. Agents Chemother. 66 (6), e0218921. 10.1128/aac.02189-21 35575578 PMC9211414

[B49] Herrmann F. R. Safran C. Levkoff S. E. Minaker K. L. (1992). Serum albumin level on admission as a predictor of death, length of stay, and readmission. Arch. Intern Med. 152 (1), 125–130. 10.1001/archinte.152.1.125 1728907

[B50] Hirsch L. Bellesoeur A. Boudou-Rouquette P. Arrondeau J. Thomas-Schoemann A. Kirchgesner J. (2020). The impact of body composition parameters on severe toxicity of nivolumab. Eur. J. cancer (Oxford, Engl. 1990) 124, 170–177. 10.1016/j.ejca.2019.11.003 31794927

[B51] Iirola T. Ihmsen H. Laitio R. Kentala E. Aantaa R. Kurvinen J. P. (2012). Population pharmacokinetics of dexmedetomidine during long-term sedation in intensive care patients. Br. J. Anaesth. 108 (3), 460–468. 10.1093/bja/aer441 22277665

[B52] Inoue K. Yuasa H. (2014). Molecular basis for pharmacokinetics and pharmacodynamics of methotrexate in rheumatoid arthritis therapy. Drug Metab. Pharmacokinet. 29 (1), 12–19. Epub 2013 Nov 26. 10.2133/dmpk.dmpk-13-rv-119 24284432

[B53] Jacqz-Aigrain E. Montes C. Brun P. Loirat C. (1994). Cyclosporine pharmacokinetics in nephrotic and kidney-transplanted chil-dren. Eur. J. Clin. Pharmacol. 47 (1), 61–65. 10.1007/BF00193480 7988626

[B54] Jensen L. Kupcova V. Arold G. Pettersson J. Hjerpsted J. B. (2018). Pharmacokinetics and tolerability of semaglutide in people with hepatic impairment. Diabetes Obes. Metab. 20 (4), 998–1005. 10.1111/dom.13186 29205786 PMC5873441

[B55] Jeon Y. J. Kim Y. S. (2011). Cyclosporin a inhibits albumin synthesis in Huh7 cells. Korean J. Intern Med. 26 (3), 314–319. 10.3904/kjim.2011.26.3.314 22016592 PMC3192204

[B56] Kawai M. Harada M. Motoike Y. Koshikawa M. Ichikawa T. Watanabe E. (2019). Impact of serum albumin levels on supratherapeutic PT-INR control and bleeding risk in atrial fibrillation patients on warfarin: a prospective cohort study. Int. J. Cardiol. Heart Vasc. 22, 111–116. 10.1016/j.ijcha.2019.01.002 30705936 PMC6348981

[B57] Keller E. Hoppe-Seyler G. Schollmeyer P. (1982). Disposition and diuretic effect of furosemide in the nephrotic syndrome. Clin. Pharmacol. Ther. 32 (4), 442–449. 10.1038/clpt.1982.187 7116760

[B58] Khera S. Sharma G. Negi V. Shaw S. C. (2022). Hypoalbuminemia and not undernutrition predicts high-dose methotrexate-induced nephrotoxicity in children with acute lymphoblastic leukemia in resource-constrained centers. Pediatr. Blood Cancer 69 (9), e29738. 10.1002/pbc.29738 35451162

[B59] Klinkmann G. Klammt S. Jäschke M. Henschel J. Gloger M. Reuter D. A. (2022). Impact of albumin binding function on pharmacokinetics and pharmacodynamics of furosemide. Med. Kaunas. 58 (12), 1780. 10.3390/medicina58121780 PMC9787406 36556982

[B60] Knudsen L. B. Lau J. (2019). The discovery and development of liraglutide and semaglutide. Front. Endocrinol. (Lausanne) 10, 155. Published 2019 Apr 12. 10.3389/fendo.2019.00155 31031702 PMC6474072

[B61] Krasowski M. D. Penrod L. E. (2012). Clinical decision support of therapeutic drug monitoring of phenytoin: measured versus adjusted phenytoin plasma concentrations. BMC Med. Inf. Decis. Mak. 12 (1), 7. 10.1186/1472-6947-12-7 PMC3312854 22333264

[B62] Lai J. C. Bikle D. D. Lizaola B. Hayssen H. Terrault N. A. Schwartz J. B. (2015). Total 25(OH) vitamin D, free 25(OH) vitamin D and markers of bone turnover in cirrhotics with and without synthetic dysfunction. Liver Int. 35 (10), 2294–2300. 10.1111/liv.12819 25757956 PMC4567539

[B63] Lee T. H. Kuo G. Chang C. H. Huang Y. T. Yen C. L. Lee C. C. (2021). Diuretic effect of co-administration of furosemide and albumin in comparison to furosemide therapy alone: an updated systematic review and meta-analysis. PLoS One 16 (12), e0260312. 10.1371/journal.pone.0260312 34851962 PMC8635380

[B64] Levitt D. G. Levitt M. D. (2016). Human serum albumin homeostasis: a new look at the roles of synthesis, catabolism, renal and gastrointestinal excretion, and the clinical value of serum al-bumin measurements. Int. J. Gen. Med. 9, 229–255. Pub-lished 2016 Jul 15. 10.2147/IJGM.S102819 27486341 PMC4956071

[B65] Li Y. Theuretzbacher U. Clancy C. J. Nguyen M. H. Derendorf H. (2010). Pharmacokinetic/pharmacodynamic profile of posaconazole. Clin. Pharmacokinet. 49 (6), 379–396. 10.2165/11319340-000000000-00000 20481649

[B66] Lindholm A. Henricsson S. Dahlqvist R. (1990). The effect of food and bile acid administration on the relative bioavailability of cyclosporin. Br. J. Clin. Pharmacol. 29, 541–548. 10.1111/j.1365-2125.1990.tb03677.x 2350530 PMC1380153

[B67] Liu J. Zhang S. Huang S. Chen Y. Zhang L. Du H. (2021). Rationality of time-dependent anti-microbial use in intensive care units in China: a nationwide cross-sectional survey. Front. Med. (Lausanne) 8, 584813. 10.3389/fmed.2021.584813 33681240 PMC7925833

[B68] Liumbruno G. M. Bennardello F. Lattanzio A. Piccoli P. Rossettias G. Italian Society of Transfusion Medicine and Immunohaematology (SIMTI) (2009). Recommendations for the use of albumin and immunoglobulins. Blood Transfus. 7 (3), 216–234. 10.2450/2009.0094-09 19657486 PMC2719274

[B69] Loh T. P. Metz M. P. (2015). Trends and physiology of common serum biochemistries in children aged 0-18 years. Pathology 47 (5), 452–461. 10.1097/PAT.0000000000000274 26126034

[B70] Mahmoodpoor A. Zahedi S. Pourakbar A. Hamishehkar H. Shadvar K. Asgharian P. (2020). Efficacy of furosemide-albumin compared with furosemide in critically ill hypoalbuminemia patients admitted to intensive care unit: a prospective randomized clinical trial. Daru 28 (1), 263–269. 10.1007/s40199-020-00339-8 32291620 PMC7214594

[B71] Maleki S. Corallo C. Coutsouvelis J. Singh J. (2018). Failure to achieve therapeutic levels with high-dose posaconazole tablets potentially due to enhanced clearance. J. Oncol. Pharm. Pract. 24 (1), 63–66. 10.1177/1078155216673228 27824587

[B72] Malluche H. H. Goldstein D. A. Massry S. G. (1979). Osteomalacia and hyperparathyroid bone disease in patients with nephrotic syndrome. J. Clin. Invest 63 (3), 494–500. 10.1172/JCI109327 429568 PMC371978

[B73] Manasa M. Beyatricks J. Martin A. Vaisakh U. (2023). Impact of hypoalbuminemia on response and toxicity ofAntineoplastic agents in patients diagnosed with solid malignancies. IJPRA 8 (Issue 4 July), 1097–1102.

[B74] Marini J. Hammond B. B. Rennebaum J. Weeda E. Misniakiewicz J. Sion A. (2022). The link between hypoalbuminemia and oral tyrosine kinase inhibitor adverse events in adults with malignant solid tumors. JHOP - August 12 (No 4).

[B75] McSorley S. T. Dolan R. D. Roxburgh C. S. D. McMillan D. C. Horgan P. G. (2017). How and why systemic in-flammation worsens quality of life in patients with advanced cancer. Expert Rev. Qual. Life Cancer Care 2 (3), 167–175. 10.1080/23809000.2017.1331705

[B76] Meenks S. D. Punt N. le Noble JLML Foudraine N. A. Neef K. Janssen P. K. C. (2023). Target attainment and population pharmacokinetics of flucloxacillin in critically ill patients: a multicenter study. Crit. Care 27 (1), 82. 10.1186/s13054-023-04353-5 36869388 PMC9982780

[B77] Mochizuki M. Murase S. Takahashi K. Shimada S. Kume H. Iizuka T. (2000). Serum itraconazole and hydroxyitraconazole concentrations and interaction with digoxin in a case of chronic hypertrophic pachymenigitis caused by Aspergillus flavus. Jpn. J. Med. Mycol. 41 (1), 33–39. 10.3314/jjmm.41.33 10660641

[B78] Murdock J. L. Duco M. R. Reeves D. J. (2021). Tolerability of highly protein bound targeted oral oncolytic drugs in patients with hypoalbuminemia: a retrospective analysis. Ann. Pharmacother. 55 (2), 165–173. 10.1177/1060028020942485 32674695

[B79] Naito M. Takei T. Eguchi A. Uchida K. Tsuchiya K. Nitta K. (2008). Monitoring of blood cyclosporine concentration in steroid-resistant nephrotic syndrome. Intern Med. 47 (18), 1567–1572. 10.2169/internalmedicine.47.1088 18797114

[B80] Nakura H. Tateishi T. Watanabe M. Asoh M. Tanaka M. Kumai T. (1998). Effect of hypoalbuminemia on the disposition of theophylline. Comparative study with Sprague-Dawley rats and a mutant Sprague-Dawley hyperlipidemic strain with hypoalbuminemia. Drug Metab. Dispos. 26 (6), 595–597. 9616197

[B81] Nishimura T. Uemura O. Hibino S. Tanaka K. Kitagata R. Yuzawa S. (2022). Serum albumin level is associated with mycophenolic acid concentration in children with idiopathic nephrotic syndrome. Eur. J. Pediatr. 181 (3), 1159–1165. 10.1007/s00431-021-04294-7 34779909

[B82] Nix D. E. Al-Obaidi M. Zangeneh T. (2024). Hypoalbuminemia and posaconazole therapeutic drug monitoring. Open Forum Infect. Dis. 11 (8), ofae452. 10.1093/ofid/ofae452 39205926 PMC11350285

[B83] Öbrink-Hansen K. Wiggers H. Bibby B. M. Hardlei T. F. Jensen K. Kragh T. (2017). Penicillin G treatment in infective endocarditis patients - does standard dosing result in therapeutic plasma concentrations? Basic Clin. Pharmacol. Toxicol. 120 (2), 179–186. 10.1111/bcpt.12661 27565458

[B84] Parfitt J. R. Jayakumar S. Driman D. K. (2008). Mycophenolate mofetil-related gastrointestinal mucosal injury: variable injury pat-terns, including graft-versus-host disease-like changes. Am. J. Surg. Pathol. 32 (9), 1367–1372. 10.1097/pas.0b013e31816bf3fe 18763324

[B85] Park H. (2011). The emergence of mycophenolate mofetilin dermatology: from its roots in the world of organ transplantation to its versatile role in the dermatology treatment room. J. Clin. Aesthet. Dermatol 4 (1), 18–27. PMC3030212 21278895

[B86] Pickup M. E. (1979). Clinical pharmacokinetics of prednisone and prednisolone. Clin. Pharmacokinet. 4 (2), 111–128. 10.2165/00003088-197904020-00004 378499

[B87] Pisupati J. Jain A. Burckart G. Hamad I. Zuckerman S. Fung J. (2005). Intraindividual and interindividual variations in the pharmacokinetics of mycophenolic acid in liver transplant patients. J. Clin. Pharmacol. 45, 34–41. 10.1177/0091270004270145 15601803

[B88] Reine P. A. Vethe N. T. Kongsgaard U. E. Andersen A. M. Line P. D. Ali Am (2013). Mycophenolate pharmacokinetics and inosine monophosphate dehydrogenase activity in liver transplant recipients with an emphasis on therapeutic drug monitoring. Scand. J. Clin. Lab. Invest 73 (2), 117–124. 10.3109/00365513.2012.745947 23281843

[B89] Reinhart Wh G. (1993). Binding of cyclosporine by erythrocytes: influence on cell shape and deformability. Eur. J. Clin. Invest 23 (3), 177–181. 10.1111/j.1365-2362.1993.tb00758.x 8477792

[B90] Reiss S. N. Buie L. W. Adel N. Goldman D. A. Devlin S. M. Douer D. (2016). Hypoalbuminemia is significantly associated with in-creased clearance time of high dose methotrexate in patients being treated for lymphoma or leukemia. Ann. Hematol. 95 (12), 2009–2015. 10.1007/s00277-016-2795-7 27542957 PMC5572815

[B91] Resztak M. Sobiak J. Czyrski A. (2021). Recent advances in therapeutic drug monitoring of voriconazole, mycophenolic acid, and vancomycin: a literature review of pediatric studies. Pharmaceutics 13 (12), 1991. 10.3390/pharmaceutics13121991 34959272 PMC8707246

[B92] Richards D. M. Heel R. C. Brogden R. N. Speight T. M. Avery G. S. (1984). Ceftriaxone. A review of its antibacterial activity, pharmacological properties and therapeutic use. Drugs 27 (6), 469–527. 10.2165/00003495-198427060-00001 6329638

[B93] Riker R. R. Gagnon D. J. Hatton C. May T. Seder D. B. Stokem K. (2017). Valproate protein binding is highly variable in ICU patients and not predicted by total serum concentrations: a case series and literature review. Pharmacotherapy 37 (4), 500–508. 10.1002/phar.1912 28173638

[B94] Roberts J. A. Pea F. Lipman J. (2013). The clinical relevance of plasma protein binding changes. Clin. Pharmacokinet. 52 (1), 1–8. 10.1007/s40262-012-0018-5 23150213

[B95] Roberts J. A. Stove V. De Waele J. J. Sipinkoski B. McWhinney B. Ungerer J. P. (2014). Variability in protein binding of teicoplanin and achievement of therapeutic drug monitoring targets in critically ill patients: lessons from the DALI Study. Int. J. Antimicrob. Agents 43 (5), 423–430. 10.1016/j.ijantimicag.2014.01.023 24630304

[B96] Rozzini L. Chilovi B. V. Bertoletti E. Ghianda D. Conti M. Trabucchi M. (2008). Serum albumin level interferes with the effect of donepezil in Alzheimer's disease. Aging Clin. Exp. Res. 20 (6), 509–512. 10.1007/BF03324877 19179833

[B97] Saito T. Iwano M. Matsumoto K. Mitarai T. Yokoyama H. Yorioka N. (2014). Significance of combined cyclosporine-prednisolone therapy and cyclosporine blood concentration monitoring for idiopathic membranous nephropathy with steroid-resistant nephrotic syndrome: a randomized controlled multicenter trial. Clin. Exp. Nephrol. 18 (5), 784–794. 10.1007/s10157-013-0925-2 24363128 PMC4194018

[B98] Sanquer S. Breil M. Baron C. Dhamane D. Astier A. Lang P. (1999). Induction of inosine monophosphate dehydrogenase activity after long-term treatment with mycophenolate mofetil. Clin. Pharmacol. Ther. 65, 640–648. 10.1016/S0009-9236(99)90085-1 10391669

[B99] Schmidt-Gayk H. Grawunder C. Tschöpe W. Schmitt W. Ritz E. Pietsch V. (1977). 25-hydroxy-vitamin-D in nephrotic syndrome. Lancet 2 (8029), 105–108. 10.1016/s0140-6736(77)90118-0 69193

[B100] Shirai S. Imai N. Sueki S. Matsui K. Tominaga N. Sakurada T. (2018). Combined cyclosporine and prednisolone therapy using cyclosporine blood concentration monitoring for adult patients with new-onset minimal change nephrotic syndrome: a single-center pilot randomized trial. Clin. Exp. Nephrol. 22 (2), 283–290. 10.1007/s10157-017-1443-4 28699032

[B101] Sjöström P. A. Odlind B. G. Beermann B. A. Karlberg B. E. (1989). Pharmacokinetics and effects of frusemide in patients with the nephrotic syndrome. Eur. J. Clin. Pharmacol. 37 (2), 173–180. 10.1007/BF00558227 2792172

[B102] Smith B. S. Yogaratnam D. Levasseur-Franklin K. E. Forni A. Fong J. (2012). Introduction to drug pharmacokinetics in the critically ill patient. Chest 141 (5), 1327–1336. 10.1378/chest.11-1396 22553267

[B103] Szałek E. Tomczak H. Smuszkiewicz P. Kamińska A. Grześkowiak E. Skóra M. (2009). Basic PK/PD parameters used in anti-microbial therapy. Anestezjol. Ratow. 3, 88–93.

[B104] Takahashi T. Terazono H. Suetsugu T. Sugawara H. Arima J. Nitta M. (2021). High-Trough plasma concentration of afatinib is associated with dose reduction. Cancers 13 (14), 3425. 10.3390/cancers13143425 34298637 PMC8305619

[B105] Takeda A. Horike K. Onoda H. Ohtsuka Y. Yoshida A. Uchida K. (2007). Benefits of cyclosporine absorption pro-filing in nephrotic syndrome: preprandial once-daily administration of cyclosporine microemulsion improves slow ab-sorption and can standardize the absorption profile. Nephrol. Carlt. 12 (2), 197–204. 10.1111/j.1440-1797.2007.00773.x 17371346

[B106] Tan H. Su W. Zhang W. Zhang J. Sattler M. Zou P. (2021). Albumin-binding domain extends half-life of glucagon-like peptide-1. Eur. J. Pharmacol. 890, 173650. 10.1016/j.ejphar.2020.173650 33049303

[B107] Tanaka H. Hiraga H. Takekuma Y. Harabayashi T. Nagamori S. Endo M. (2020). Possibility for dose optimization of pazopanib from its plasma concentration in Japanese patients with cancer. Biol. Pharm. Bull. 43 (5), 762–766. 10.1248/bpb.b19-00560 32115446

[B108] Tbler S. M. Wilkerson M. A. Porter W. H. Smith A. J. Chandler M. H. H. (1990). Severe phenytoin intoxication as a result of altered protein binding in aids. DICP 24 (7-8), 698–700. 10.1177/106002809002400708 2375139

[B109] Tincani E. Mazzali F. Morini L. (2002). Hypoalbuminemia as a risk factor for over-anticoagulation. Am. J. Med. 112 (3), 247–248. 10.1016/s0002-9343(01)00957-3 11893361

[B110] Tobler A. Hösli R. Mühlebach S. Huber A. (2016). Free phenytoin assessment in patients: measured versus calculated blood serum levels. Int. J. Clin. Pharm. 38 (2), 303–309. 10.1007/s11096-015-0241-x 26746902

[B111] Toh Y. L. Pang Y. Y. Shwe M. Kanesvaran R. Toh C. K. Chan A. (2020). HPLC‐MS/MS coupled with equilibrium dialysis method for quantification of free drug concentration of pazopanib in plasma. Heliyon 6 (4), e03813–‐. 10.1016/j.heliyon.2020.e03813 32373731 PMC7191207

[B112] Traeger S. M. Haug M. T. (1986). Reduction of diazepam serum half life and reversal of coma by activated charcoal in a patient with severe liver disease. J. Toxicol. Clin. Toxicol. 24 (4), 329–337. 10.3109/15563658608992597 3746988

[B113] Treon S. P. Chabner B. A. (1996). Concepts in use of high dose methotrexate therapy. Clin. Chem. 42, 1322–1329. 10.1093/clinchem/42.8.1322 8697606

[B114] Van den Ouweland F. A. Gribnau F. W. Tan Y. Van Ginneken C. A. Van de Putte L. B. (1986). Hypoalbuminaemia and naproxen pharmacokinetics in a patient with rheumatoid arthritis. Clin. Pharmacokinet. 11 (6), 511–515. 10.2165/00003088-198611060-00007 3802691

[B115] Van den Ouweland F. A. Gribnau F. W. Van Ginneken C. A. Tan Y. Van de Putte L. B. (1988). Naproxen kinetics and disease activity in rheumatoid arthritis: a within-patient study. Clin. Pharmacol. Ther. 43 (1), 79–85. 10.1038/clpt.1988.14 3335121

[B116] van Hoof H. J. de Sévaux R. G. van Baelen H. Swinkels L. M. Klipping C. Ross H. al (2001). Relationship between free and total 1,25-dihydroxyvitamin D in conditions of modified binding. Eur. J. Endocrinol. 144 (4), 391–396. 10.1530/eje.0.1440391 11275949

[B117] Vega G. L. Grundy S. M. (1988). Lovastatin therapy in nephrotic hyperlipidemia: effects on lipoprotein metabolism. Kidney Int. 33 (6), 1160–1168. 10.1038/ki.1988.125 3165483

[B118] Wang J. Zeevi A. Webber S. Girnita D. M. Addonizio L. Selby R. (2007). A novel variant L263F in human inosine 5 ’ -monophosphate dehydrogenase 2 is associated with diminished enzyme activity. Pharmacogenet Genomics 17, 283–290. 10.1097/FPC.0b013e328012b8cf 17496727

[B119] Weinblatt M. E. (1989). Drug interactions with non-steroidal anti-inflammatory drugs (NSAIDs). Scand. J. Rheumatol. Suppl. 83, 7–10. 10.3109/03009748909101471 2576330

[B120] Wright J. D. Boudinot F. D. Ujhelyi M. R. (1996). Measurement and analysis of unbound drug con-centrations. Clin. Pharmacokinet. 30 (6), 445–462. 10.2165/00003088-199630060-00003 8792057

[B121] Xu H. Liu Q. (2021). Individualized management of blood concentration in patients with gastrointestinal stromal tumors. Onco Targets Ther. 13, 13345–13355. Published 2021 Jan 5. 10.2147/OTT.S279998 33456310 PMC7804055

[B122] Yu L. Hua Z. Luo X. Zhao T. Liu Y. (2022). Systematic interaction of plasma albumin with the efficacy of chemotherapeutic drugs. Biochim. Biophys. Acta Rev. Cancer 1877 (1), 188655. 10.1016/j.bbcan.2021.188655 34780933

[B123] Zarowitz B. Shlom J. Eichenhorn M. S. Popovich J. Jr (1985). Alterations in theophylline protein binding in acutely ill patients with COPD. Chest 87 (6), 766–769. 10.1378/chest.87.6.766 3996064

[B124] Zhang T. Deng Y. He P. He Z. Wang X. (2015). Effects of mild hypoalbuminemia on the pharmacokinetics and pharmacodynamics of dexmedetomidine in patients after major abdominal or thoracic surgery. J. Clin. Anesth. 27 (8), 632–637. 10.1016/j.jclinane.2015.06.002 26277872

[B125] Zhao W. Elie V. Baudouin V. Bensman A. André J. L. Brochard K. al (2010). Population pharmacokinetics and Bayesian estimator of mycophenolic acid in children with idiopathic nephrotic syndrome. Br. J. Clin. Pharmacol. 69 (4), 358–366. 10.1111/j.1365-2125.2010.03615.x 20406220 PMC2848409

